# Coumaric acid-induced spontaneous gelation of apple pectin with controllable structure and multifunctional properties

**DOI:** 10.1016/j.crfs.2026.101443

**Published:** 2026-05-18

**Authors:** Lanlan Hu, Yifei Bai, Guanglei Li, Ibrahim Khalifa, Benguo Liu, Hao Zhang, Yangyang Jia

**Affiliations:** a School of Food Science, Henan Institute of Science and Technology, Xinxiang, 453003, China; b Shaanxi Research Institute of Agricultural Products Processing Technology, Xi'an, 710021, China; c Food Technology Department, Faculty of Agriculture, Benha University, Moshtohor, 13736, Egypt; d Department of Food Science, College of Agriculture and Veterinary Medicine, United Arab Emirates University, Al-Ain, 15551, United Arab Emirates

**Keywords:** Apple pectin, Coumaric acid, Interaction, Spontaneous gelation, Composite hydrogel, Rheological property

## Abstract

This study investigates the ability of p-coumaric acid (CA) to induce spontaneous gelation of low-methoxyl apple pectin (AP, DM 30%–40%) under sucrose-free, calcium-free, and heat-free conditions at pH 3.0, and characterize the structural, mechanical, and functional properties of the resulting composite hydrogels. Stable elastic-dominant hydrogels were formed at CA concentrations of 4–9 mg/mL, with a precisely defined critical gelation concentration of 4.0 mg/mL. Rheological measurements revealed that the storage modulus (*G′*) increased from 0.48 ± 0.02 Pa in AP dispersion to 13.92 ± 0.10 Pa in composite hydrogels containing 9 mg/mL CA, accompanied by pronounced shear-thinning behavior and syringeability with long-term shape retention. The hydrogels exhibited tunable water retention and freeze–thaw stability, with CA incorporation significantly restricting water mobility in the network. Meanwhile, the composite hydrogels possessed robust antioxidant activity (DPPH: ∼90%; ABTS: >95%), broad-spectrum antibacterial effects against both Gram-positive and Gram-negative strains, and favorable growth compatibility with *Lactobacillus plantarum*even at the highest tested CA concentration (9 mg/mL). Multi-scale characterization and molecular docking indicated that hydrogen bonding between AP and CA was the primary drivers for network formation, with CA-mediated shielding of interchain electrostatic repulsion acting as a prerequisite for the ordered spontaneous self-assembly of AP chains. This work provides a novel, mild, and clean-label strategy to develop multifunctional AP–based hydrogels with controllable viscoelasticity and bioactivity, highlighting great application potential in food, biomedical, and packaging fields.

## Highlights

• p-Coumaric acid induces spontaneous apple pectin gelation, free of sucrose, calcium, or heat.
• Hydrogels show tunable viscoelasticity, shear-thinning behavior, and excellent syringeability.
• The system exhibits robust antioxidant, antibacterial effects, and probiotic compatibility.
• Hydrogen bonding dominates gel formation, with electrostatic shielding as a prerequisite for self-assembly.

## Graphical abstract

**Figure:**
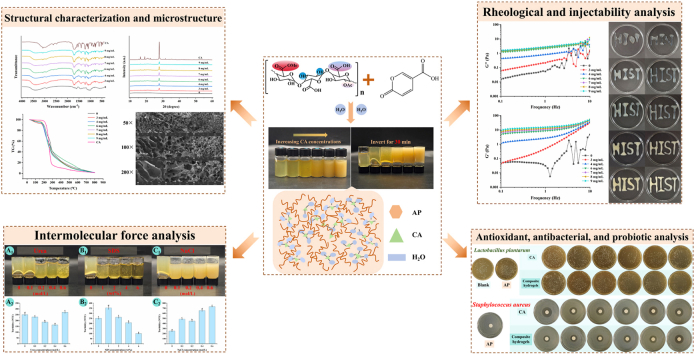

## 1 Introduction

Pectin, a structurally complex acidic polysaccharide abundant in the middle lamella of higher plants, has attracted considerable attention in the food, pharmaceutical, and cosmetic industries owing to its excellent biocompatibility, biodegradability, and versatile functional properties (Feng et al., 2023; Petkowicz and Williams, 2020). Structurally, pectin consists of three major domains: linear homogalacturonan (HG), composed of α-1,4-linked D-galacturonic acid residues, branched rhamnogalacturonan I (RG-I) with repeating galacturonic acid-rhamnose disaccharide backbones, and highly complex rhamnogalacturonan II (RG-II) (Yang et al., 2018; Zhu et al., 2024). Based on the degree of methylation (DM), pectin is categorized into high-methoxyl pectin (HMP, DM > 50%) and low-methoxyl pectin (LMP, DM < 50%) (Mamet et al., 2017). Both types are widely used as gelling, thickening, and emulsifying agents, but their gelation mechanisms are fundamentally distinct: HMP forms gels under acidic (pH 2.8–3.6) and high sucrose concentrations (>55%) through hydrogen bonding and hydrophobic associations, whereas LMP relies on divalent cations such as Ca^2+^ to establish ionic cross-links (Feng et al., 2023). However, these conventional gelation strategies have notable limitations: (i) dependence on high sugar or cation levels, which is incompatible with the growing demand for low-sugar, clean-label food systems; (ii) strict requirements for pH and ion conditions that restrict formulation flexibility; and (iii) risks of phase separation and textural instability during long-term storage. Consequently, there is an urgent need to develop novel, sustainable pectin gelation strategies that avoid these constraints, with no need for exogenous additives or harsh processing conditions, while maintaining tunable mechanical strength and functional stability.

Pectin gelation is essentially the formation of a three-dimensional (3D) polymeric network stabilized by non-covalent interactions including hydrogen bonds, hydrophobic associations, and electrostatic forces (Yang et al., 2018). The mechanical and functional properties of the resulting gels are governed by polymer structure, chain conformation, and the nature of intermolecular junction zones (Maiz-Fernández et al., 2021; Tong et al., 2024). Recently, non-covalent self-assembly has been identified as a sustainable route for hydrogel formation, as it avoids chemical cross-linkers and operates under mild, ambient conditions, making it suitable for food and nutraceutical applications (Kazemi-Taskooh and Varidi, 2021). Polyphenols, a large family of plant-derived bioactive compounds, have been widely investigated as effective modulators of polysaccharide gelation, owing to their ability to interact with polysaccharide chains (Ma et al., 2024). Previous studies have demonstrated that polyphenols can bind with pectin *via* hydrogen bonds and hydrophobic interactions, modifying chain conformation, rheological properties, and gelation behavior. For example, Mamet et al. (2017) reported that persimmon tannins can enhance the gel strength of both HMP and LMP in traditional gelation systems; Ahn et al. (2017) developed pectin–polyphenol conjugates with antioxidant activity, which still required ion-induced gelation to form hydrogels. Beyond pectin systems, spontaneous polyphenol–induced polysaccharide gelation has been reported in other matrices, such as tea polyphenol–triggered gelation of *Nicandra physalodes* seed polysaccharides (Ma et al., 2024) and tannic acid–induced gelation of quaternized chitin (Xie et al., 2025). However, most existing pectin–polyphenol systems still rely on covalent modification, additional cross-linking agents, or traditional gelation conditions (cations, high sugar, thermal treatment). This represents a major research gap in the design of low-sugar, natural pectin gels. Spontaneous gelation of native pectin induced solely by non-covalent interactions with polyphenols, without any exogenous additives or harsh processing conditions, remains poorly understood and has not been systematically established, representing a key research gap for the development of clean-label pectin-based functional materials.

To bridge this gap, a systematic screening of common phenolic acids with diverse structural features was first performed, including caffeic acid, ferulic acid, gallic acid, protocatechuic acid, chlorogenic acid, and ellagic acid—all of which have been widely reported to interact with polysaccharides in previous studies. Screening results confirmed that none of these phenolic acids could induce spontaneous gelation of apple pectin (AP) under sugar-free, cation-free, and ambient temperature conditions. Notably, p-coumaric acid (CA), a hydroxycinnamic acid widely present in fruits and vegetables, has a unique structural feature: a single phenolic hydroxyl group on the phenyl ring and a conjugated C=C–COOH moiety (Derebasi et al., 2024; Huang et al., 2023). This structure enables moderate and specific non-covalent interactions with pectin chains: hydrogen bonding between the phenolic hydroxyl/carboxyl groups of CA and the hydroxyl/carboxyl groups of pectin, as well as hydrophobic associations between the aromatic ring of CA and the hydrophobic domains of the pectin backbone. Such moderate intermolecular interactions can promote the ordered self-assembly of pectin chains into a stable, homogeneous network, without causing over-aggregation or precipitation, which cannot be achieved by other tested phenolic acids. This provides a clear rationale for the selection of CA as the gelation inducer in this work.

Accordingly, this study aims to develop a novel, spontaneous, additive-free gelation strategy for native pectin induced solely by CA *via* non-covalent self-assembly. The specific research objectives are: (i) to characterize the gelation behavior and microstructure features of CA–induced AP hydrogels; (ii) to evaluate the physicochemical, rheological, and functional properties of the hydrogels, including water retention, freeze–thaw stability, antioxidant, antibacterial activities, and probiotic compatibility; (iii) to elucidate the molecular mechanisms and non-covalent forces governing the spontaneous self-assembly and gel process. Compared with established pectin-polyphenol systems, this work realizes spontaneous room-temperature gelation of native pectin solely *via* CA-mediated interactions, free of exogenous sugar, cations or thermal treatment. The resulting hydrogel, with tunable mechanics, robust bioactivity and favorable probiotic compatibility, provides a new platform for clean-label functional foods and nutraceuticals.

## 2 Materials and methods

### 2.1 Materials and chemicals

Apple-derived pectin (DM: 30%-40%, Mw: 323108 Da; CAS: 9000-69-5, Product No. P885045) and p-coumaric acid (purity >97%, CAS: 501-98-4, Product No. C804995, derived from sugarcane by-products) were obtained from Macklin Biochemical Technology Co., Ltd (Shanghai, China). The molecular weight information of AP was supplemented in Fig. S1 and Table S1. Sodium chloride (NaCl, CAS: 7647-14-5), potassium bromide (KBr, CAS: 7758-02-3), urea (CAS: 57-13-6), and sodium dodecyl sulfate (SDS, CAS: 151-21-3) were obtained from Sinopharm Chemical Reagent Co., Ltd (Shanghai, China). De Man, Rogosa and Sharpe (MRS, Product No. HB0384-1) broth was supplied by Haibo media (Qingdao, China). All other chemicals used were of analytical grade unless otherwise specified.

### 2.2 Preparation of AP–CA composite hydrogels

Samples were prepared in 0.1 M sodium citrate buffer (pH 3.0) to maintain a uniform acidic environment during gel formation. AP powder was dispersed in the buffer to obtain a homogeneous 10 mg/mL solution, followed by stirring at 500 rpm for 30 min to ensure complete hydration. CA was subsequently added to the AP dispersion to achieve final concentrations of 0, 0.2, 0.5, 1, 2, 3, 4, 5, 6, 7, 8, 9, 10, and 15 mg/mL. The pH of each mixture was measured before and after CA addition, with all samples remaining within ±0.1 pH units, indicating negligible pH fluctuation during sample preparation. The mixtures were stored at 4 °C for 2 h to reach reaction equilibrium, with measures taken to prevent syneresis and water evaporation prior to further testing. The solubility of CA in aqueous buffer system was pre-evaluated to guide the selection of effective CA concentrations for gel formation (Fig. S2). CA maintained 100% solubility at concentrations ≤3 mg/mL, while solubility decreased gradually with further elevation of CA concentration. This solubility curve served as the core basis for concentration gradient design in subsequent gelation screening, to avoid interference from CA precipitation on the evaluation of gelation behavior.

### 2.3 Visual appearance and color evaluation

Hydrogel samples were placed in culture dishes and photographed every two days over a 15-day period. Color parameters (*L*∗, *a*∗, and *b*∗) were measured with a colorimeter (Konica-Minolta, CR-400, Osaka, Japan), and chroma (*C*∗) and chromaticity difference (Δ*E*∗) were calculated to comprehensively evaluate color changes. Measurements were taken at six random positions per sample, and the mean values were reported.

### 2.4 Rheological and texture analysis

Rheological tests (strain and frequency sweeps) were performed to obtain key viscoelastic parameters (*G′*, *G″*, and tan *δ*). Combined analysis of these data was used to elucidate the structure–function relationship of the hydrogels' dynamic mechanical performance. The mechanical properties of composite hydrogels were determined using texture profile analysis (TPA) and rheometry. TPA yielded four core texture parameters (hardness, cohesiveness, springiness, chewiness), which reflect the hydrogel's network compactness, gel strength, and structural stability.

#### 2.4.1 Rheological analysis

The rheological properties of the hydrogels were characterized using a Rheometer (HAAKE MARS III, Thermo Fisher Scientific, Karlsruhe, Germany) equipped with a 60 mm parallel plate. A sample gap of 1.0 mm was used, and the plate rim was sealed with a thin layer of silicone oil to prevent evaporation.

Strain sweep tests were performed at a constant frequency of 1 Hz with oscillatory strain ranging from 0.01% to 100%. The storage modulus (*G′*) was plotted as a function of strain to determine the linear viscoelastic region (LVR). The initial strain corresponds to the lowest measured strain, while the critical strain is defined as the point at which *G′* deviates by 10% from the plateau. The crossover strain, where *G′* = loss modulus (*G″*), was also identified.

Frequency sweep tests were conducted within the LVR at 25 °C, covering a frequency range of 0.1–10 Hz. The frequency-dependent *G′*, *G″*, and loss angle (tan *δ* = *G″*/*G′*) were recorded to evaluate hydrogel strength, interaction lifetime, and gel type. Mechanical spectra were fitted using the Power Law model:

*G′ = K'ω*^*n*'^, *G″ = K″ω*^*n*^*″*, and *G∗ = A*_*α*_*ω*^*α*^

where *K′* and *K″* are the constants, *n'* and *n"* are frequency exponents, *A*_*α*_ is the gel network strength (Pa·s^*α*^), *α* is the relaxion order, and *ω* is the angular frequency (Li et al., 2023; Wang et al., 2022).

Apparent viscosity and shear stress were measured at shear rates ranging from 0.1 to 100 s^−1^. The flow behavior was analyzed from apparent viscosity–shear rate curves. Shear stress–shear rate data were further fitted to the Power Law model:

$$\sigma =K\times {ṙ}^{n}$$

where *σ* is the shear stress (Pa), *γ* is the shear rate (s^−1^), *K* is the consistency coefficient (Pa·s^n^), and *n* is the flow behavior index.

#### 2.4.2 Texture measurement

TPA tests of the composite hydrogels were performed using a TA-XT Plus Texture Analyzer (Stable Micro Systems Ltd., Godalming, UK). Samples were compressed to 40% deformation using a P/36R plunger, with a trigger force of 5.0 g. Two consecutive compression cycles were applied for each sample, with a compression speed of 1.0 mm/s and relaxation speed of 5.0 mm/s. Key texture parameters were defined and calculated *via* Exponent software as follows: (i) Hardness, maximum force recorded in the first compression cycle, directly reflecting hydrogel strength and resistance to external deformation. (ii) Springiness: Ratio of sample height recovered after the first compression to the initial deformation, representing the elastic recovery capacity of the hydrogel network. (iii) Cohesiveness: Ratio of the positive force area of the second compression cycle to that of the first, quantifying the internal binding strength of the gel structure against continuous damage. (iv) Chewiness: reflecting the total energy required to disrupt the hydrogel matrix. All measurements were performed in quintuplicate.

### 2.5 Turbidity and transparency measurement

Turbidity was measured using a HACH 2100 N Laboratory Turbidimeter (HACH, Loveland, USA) equipped with a tungsten-filament lamp and three detectors (90° scattered-light, forward-scatter, and transmitted light). Calibration was performed using a Gelex® Secondary Turbidity Standard Kit (HACH, Loveland, USA). The transparency of hydrogels was determined at 650 nm using a UV–Vis spectrophotometer (TU-1810PC, PERSEE, China). Transmittance (T%) was first calibrated to 100% using distilled water as the blank. Sample transparency was then expressed as 100-T%.

### 2.6 Injectability evaluation

The injectability of hydrogel formulations was evaluated to characterize extrusion behavior and shape retention following deposition. Two sample types including AP dispersion and AP–CA composite hydrogels were tested. Each sample was loaded into a 5 mL (coarse) or 1 mL (fine) syringe equipped with standard conical tips, and manual extrusion was performed at room temperature onto a flat glass plate to deposit a predefined "HIST" pattern. Immediately after extrusion, the structures were photographed and visually examined for shape fidelity, edge sharpness, and overall structural integrity. Qualitative assessment of syringeability was conducted across two core dimensions during and after extrusion. For intra-extrusion performance, the ease and smoothness of manual extrusion through the syringe tip were qualitatively assessed as key indicators, with evaluation criteria including the absence of nozzle clogging, consistency of material flow, and required extrusion force. For post-extrusion stability, immediate visual inspection was performed on the deposited constructs after extrusion, with assessment indicators covering the sharpness of pattern edges, structural continuity, and the absence of pattern merging, distortion or collapse. Immediately after extrusion, all structures were photographed and visually examined for overall shape fidelity and structural integrity. To further characterize post-extrusion long-term stability, the injectable hydrogel structures were stored under ambient conditions for 14 days, with daily photographic monitoring of geometric and surface morphology changes to assess deformation resistance and self-supporting capability. These measurements provided practical macroscopic evidence linking the viscoelastic network integrity of AP–CA composite hydrogels to their injectable performance and long-term structural retention.

### 2.7 Water holding capacity (WHC) and syneresis rate (SR) evaluation

WHC and SR were used to link macro-level functional stability with microstructural integrity and molecular interactions. WHC of the hydrogels was determined *via* centrifugation. Briefly, fresh hydrogel samples were weighed (*M*_*b*_) and placed in centrifuge tubes. Samples were centrifuged at 10,000 × g for 20 min at 25 °C. The supernatant was carefully removed, and the remaining hydrogel was weighed (*M*_*a*_). WHC was calculated as following:

$$WHC\;\left(\%\right)=\frac{M_{a}}{M_{b}}\times 100$$

*SR* of the hydrogels was evaluated after a freeze–thaw cycle following a previously reported method (Ma et al., 2024). After the cycle, released liquid was removed, and the remaining hydrogel was weighed (*M*_*c*_). *SR* was calculated as following:

$$SR\;\left(\%\right)=\frac{M_{d}‐M_{c}}{M_{d}}\times 100$$

where *M*_*d*_ is the mass of the hydrogel before freeze–thaw treatment.

### 2.8 Low-field nuclear magnetic resonance (LF-NMR)

The water mobility in the hydrogel samples was analyzed using a MesoMR23-060H–I low-field NMR analyzer (Niumag Co., Ltd., Shanghai, China) equipped with a 0.5 T permanent magnet. The *T*_*2*_ relaxation times obtained from Carr-Purcell-Meiboom-Gill (CPMG) pulse sequences were analyzed to quantify the bound, immobilized, and free water fractions, providing molecular-level insight into the water–pectin–CA interactions and their impact on hydrogel texture and water-holding capacity. Measurement parameters were set as follows: spectral width = 100 kHz, time wait = 5000 ms, regulate first data = 0.08 ms, regulate analog gain 1 = 20, regulate digital gain 1 = 3, number sampling = 4, data radius = 1, number of echoes = 18000, echo time = 0.8 ms.

### 2.9 Antioxidant and antibacterial evaluation

The antioxidant activities of AP, CA, and AP–CA composite hydrogels were assessed by their free radical scavenging abilities against 2,2-diphenyl-1-picrylhydrazyl (DPPH) and 2,2′-azino-bis(3-ethylbenzothiazoline-6-sulfonic acid) (ABTS) radicals. Hydrogel samples were incubated with freshly prepared DPPH and ABTS radical solutions in the dark at room temperature. The absorbance was measured at 517 nm (DPPH) and 734 nm (ABTS) using a UV–Vis spectrophotometer (TU-1810PC, PERSEE, China).

For the DPPH assay, 0.4 mL of 0.2 mM DPPH ethanol solution was mixed with 0.1 mL of aqueous sample solution (concentrations: 0.125, 0.25, 0.5, 1.0, 1.5, and 2.0 mg/mL) and 0.3 mL of distilled water. The mixture was vigorously shaken and incubated at room temperature for 30 min. The DPPH radical scavenging activity was calculated as following:

$$DPPH\;radical\;scavenging\;activity\;\left(\%\right)=\left(1\;‐\;\frac{A_{s}\;‐\;A_{1}}{A_{0}}\right)\times 100$$

where *A*_*s*_ is the absorbance of the sample, *A*_*1*_ is the absorbance of the sample blank (distilled water instead of DPPH solution), and *A*_*0*_ is the absorbance of the control blank (distilled water instead of the sample).

Stable ABTS radicals were generated by oxidizing 2 mL of 7.0 mM ABTS with 2 mL of 5.0 mM potassium persulfate in the dark at room temperature for 12 h. For the ABTS assay, 0.3 mL of sample solutions at various concentrations was mixed with 3 mL of the ABTS working solution, and the absorbance was measured at 734 nm. The ABTS radical scavenging activity was calculated as following:

$$ABTS\;radical\;scavenging\;activity\;\left(\%\right)=\left(1\;‐\;\frac{A_{s}\;‐\;A_{1}}{A_{0}}\right)\times 100$$

where *A*_*s*_ is the absorbance of the sample, *A*_*1*_ is the absorbance of the sample blank (PBS buffer instead of ABTS working solution), and *A*_*0*_ is the absorbance of the control blank (distilled water instead of the sample).

The antibacterial efficacy of AP, CA, and AP–CA composite hydrogels was assessed against seven bacterial strains: *Escherichia coli*, *Staphylococcus aureus*, *Pseudomonas aeruginosa*, *Salmonella typhimurium*, *Salmonella enterica*, *Bacillus cereus*, and *Listeria monocytogenes*. All strains were first activated to reach the logarithmic growth phase, and the bacterial suspensions were adjusted to an OD_600_ of 0.02 prior to use. Subsequently, 100 μL aliquots of each suspension were spread evenly onto LB agar plates. Sterile filter paper discs (6 mm in diameter) were placed at the center of each plate, and 100 μL of each sample was applied onto the discs. The plates were then incubated at 37 °C overnight. Following incubation, the antibacterial activity was determined by measuring the diameters of the inhibition zones using a digital caliper. The functional activities were correlated with CA incorporation and hydrogel crosslink density to elucidate how structural assembly enhances antioxidant and antibacterial functions.

### 2.10 Microstructure observation

The network structures of freeze-dried hydrogels were examined using a scanning electron microscope (TESCAN MIRA LMS, Czech Republic). Samples were fixed onto conductive adhesive, mounted in a sample column, and sputter-coated with gold according to the manufacturer's instructions. Imaging was performed at an accelerating voltage of 3.0 kV at room temperature. Quantitative analysis was conducted using AngioTool, with three parameters (vessel percentage area, total number of junctions, and mean lacunarity) employed to characterize pore abundance, network connectivity, and structural uniformity, respectively.

### 2.11 Thermogravimetric analysis

Approximately 3 mg of freeze-dried samples were placed in an Al_2_O_3_ crucible and analyzed using a thermogravimetric analyzer (PerkinElmer STA 8000, USA). Measurements were performed under the flowing N_2_ atmosphere with a heating rate of 20 °C/min from 30 to 800 °C.

### 2.12 X-ray diffraction analysis

The crystallinity of freeze-dried samples was determined with a Rigaku Smartlab SE X-ray diffractometer (Rigaku, Osaka, Japan). Data were collected over a 2θ range of 10°–60° at a scan rate of 2°/min. The diffraction patterns were used to assess molecular ordering and phase transitions induced by CA incorporation. Variations in peak intensity and width were interpreted as indicators of the degree of structural regularity and the extent of CA–pectin complexation, providing insight into how CA affects the microstructural organization and rigidity of the hydrogel network.

### 2.13 Fourier transform infrared spectroscopy (FT-IR)

Freeze-dried samples (1 mg) were mixed with potassium bromide (KBr) and pressed into a transparent pellets. FT-IR spectra were recorded on a Thermo Nicolette 6700 spectrophotometer (Thermo Fisher Scientific, MA, USA) in the4000-400 cm^−1^ region at a resolution of 4 cm^−1^ with 64 scans. FT-IR analysis was conducted to detect characteristic shifts in functional groups, particularly those associated with O–H, C=O, and C–O–C stretching vibrations, to elucidate hydrogen bonding and esterification interactions between CA and AP chains. These spectral variations were used to verify molecular-level interactions that contribute to network formation and mechanical reinforcement in the CA–AP composite hydrogel.

### 2.14 Molecular forces involved in AP–CA composite hydrogel formation

To elucidate the molecular forces governing CA-induced gelation of AP, three dissociation agents targeting specific molecular forces were employed: urea (0–0.6 mol/L) for hydrogen bonding, NaCl (0–0.6 mol/L) for electrostatic interactions, and sodium dodecyl sulfate (SDS, 0–6 wt%) for hydrophobic interactions. Each dissociating reagent was pre-dissolved in 0.1 M sodium citrate buffer (pH 3.0) to ensure consistent ionic strength and pH conditions. Pre-formed AP–CA hydrogels were carefully transferred to glass vials, and the dissociating solutions were added to minimize mechanical disruption. The mixtures were allowed to react for 0.5 h at room temperature, enabling sufficient penetration of the dissociating agents into the gel network. After treatment, the degree of gel weakening or disintegration was assessed through visual observation, turbidity measurements, and changes in gel integrity. The extent of gel collapse induced by each agent provided qualitative evidence of the relative contribution of hydrogen bonding, electrostatic interactions, and hydrophobic associations to the network stabilization. These results helped clarify the structure–function relationships governing CA-induced gel assembly and the physicochemical robustness of the resulting composite hydrogels.

### 2.15 Molecular docking

Molecular docking is a widely used computational approach to predict the binding mode and interaction affinity between small molecule ligands and macromolecular receptors, which provides insights into intermolecular recognition *via* binding conformation simulation and binding free energy calculation (Senan et al., 2025; Uslu et al., 2025). In this study, the three-dimensional structure of pectin fragment was constructed using GLYCAM-Web (https://glycam.org/), a widely used tool for generating low-energy polysaccharide structures (Grant et al., 2025). The pectin sequence used was GalA-α(1–2)-[Gal-β(1–4)_9_-Gal-α(1–4)]Rha-α(1–4)-GalA-α(1–2)-[Ara-α(1–3)_9_][Gal-β(1–4)_4_-Gal-α(1–4)]Rha-α(1–4)-GalA-α(1–2)-[Ara-α(1–3)_10_][Gal-β(1–4)_4_-Gal-α(1–4)]Rha-α(1–4)-GalA-α(1–2)-[Gal-β(1–4)_9_-Gal-α(1–4)]Rha-α(1–4)-GalA (Xu et al., 2025). This sequence retains the core glycosidic bond linkage, sugar residue composition, and branched structure characteristics of native AP, providing a representative and simplified molecular model for docking simulation.

The molecular docking procedure was performed using AutoDockTools 1.5.6 and AutoDock Vina (Eberhardt et al., 2021; Trott and Olson, 2010). The structure of CA ligand was retrieved from the PubChem database (https://pubchem.ncbi.nlm.nih.gov/), followed by geometry optimization and energy-minimization using the MMFF94 force field to obtain an optimized geometry. The ligand was then converted into a pdbqt file in AutoDockTools 1.5.6 for subsequent docking. A grid box was defined around the pectin structure, and docking was carried out using AutoDock Vina to evaluate binding free energies. Visualization and interaction analysis of the resulting complexes were conducted using PyMOL (https://pymol.org/2/) and LigPlot+. PyMOL was used to inspect the three-dimensional structures of the complexes and determine the spatial orientation of CA on the pectin fragment surface. LigPlot+ was employed to generate two-dimensional interaction diagrams, enabling quantitative assessment of hydrogen bonds and hydrophobic interactions within complexes and providing a structural basis for interpreting binding affinities.

### 2.16 Statistical analysis

All experiments were performed using three independently prepared batches of samples to ensure reproducibility, unless otherwise specified. Results are presented as means ± standard deviation. Data were analyzed using one-way analysis of variance (ANOVA) using the SPSS 22.0 software package (SPSS Inc., Chicago, USA). Significant differences between means (*p* < 0.05) were determined using the Duncan multiple-range test. Figures were plotted using Origin 2021 (OriginLab, MA, USA).

## 3 Results and discussion

### 3.1 Screening of p-coumaric acid (CA) concentrations for inducing spontaneous gelation of apple pectin (AP)

**Fig. 1:**
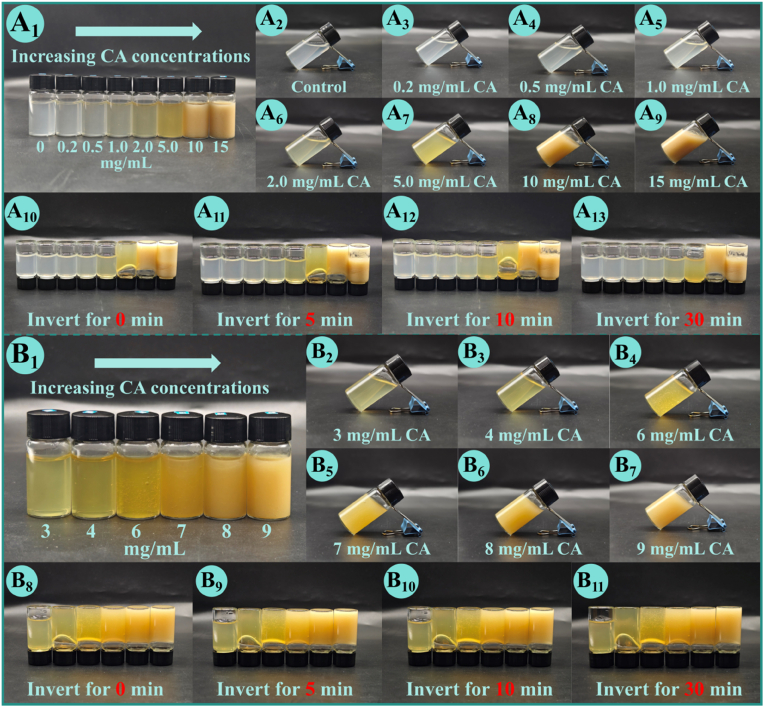
Visual appearance of AP–CA composite hydrogels at different CA concentrations. (**A**) Hydrogels prepared with CA concentrations ranging from 0 to 15 mg/mL: (**A_1_**) normal placement, (**A_2_–A_9_**) tilted view, and (**A_10_–A_13_**) inverted for 0, 5, 10, and 30 min; (**B**) Hydrogels prepared with CA concentrations of 3–9 mg/mL: (**B_1_**) normal placement, (**B_2_–B_7_**) tilted view, and (**B_8_–B_11_**) inverted for 0, 5, 10, and 30 min.

To assess the gelation-inducing capacity of CA on AP (10 mg/mL), wide-range concentration screening was initially performed with final CA concentrations of 0.2, 0.5, 1, 2, 5, 10, and 15 mg/mL (Fig. 1A). Gelation behavior was qualitatively evaluated by tube tilting at 45° (Fig. 1A_2_-A_9_) and inversion tests (Fig. 1A_10_-A_13_) at 0, 5, 10, and 30 min, to monitor macroscopic flow characteristics and mechanical stability. The control (AP dispersion) and mixtures with low CA levels (0.2, 0.5, 1, and 2 mg/mL) remained fully fluid with no self-supporting behavior, indicating the absence of gel formation. In contrast, the mixture with 5 and 10 mg/mL CA exhibited a non-flowing, gel-like state, demonstrating the effective gelation-inducing effect of CA at appropriate concentrations. Notably, partial precipitation was observed at 10 mg/mL CA, and abundant insoluble aggregates appeared in the 15 mg/mL CA sample, which is attributed to the limited solubility of CA at high concentrations (Fig. S2). To preliminarily narrow the gelation threshold, intermediate CA concentrations (3, 4, 6, 7, 8, and 9 mg/mL) were further examined (Fig. 1B). Stable, non-fluid gels were formed in mixtures with 4–9 mg/mL CA, while the 3 mg/mL CA sample remained flowable after prolonged inversion, with no stable gel formation observed. To address the sharp gelation transition between 2 and 4 mg/mL and precisely define the critical gelation concentration (CGC) of the system, fine-gradient concentration screening was supplemented for CA concentrations ranging from 2.0 to 4.0 mg/mL at 0.2 mg/mL intervals (2.0, 2.2, 2.4, 2.6, 2.8, 3.0, 3.2, 3.4, 3.6, 3.8, 4.0 mg/mL), with results presented in Fig. S3 (2.0–3.0 mg/mL) and Fig. S4 (3.0–4.0 mg/mL). Consistent with preliminary screening results, all samples with CA concentrations from 2.0 to 3.8 mg/mL remained fully fluid with no stable gel formation, while a self-supporting, non-flowing hydrogel was first observed at 4.0 mg/mL CA. This result precisely defines the CGC of CA-induced AP spontaneous gelation as 4.0 mg/mL under the current experimental conditions. The concentration-dependent gelation behavior of the AP–CA system can be elucidated *via* CA solubility, critical aggregation concentration (CAC), and intermolecular interaction mechanisms. At CA concentrations below 4 mg/mL, despite 100% solubility of CA, the quantity of CA molecules is insufficient to reach the CAC required for the formation of a continuous network. The near-"on/off" sol-gel transition at the CGC is driven by the threshold effect of intermolecular non-covalent interactions at the molecular level. Once CA concentration reaches the CGC, cross-linking sites provided by CA reach the critical threshold required for 3D network formation, and the rapid establishment of a continuous cross-linked network drastically restricts aqueous phase flow, triggering the abrupt sol-gel transition. With CA concentration exceeding 9 mg/mL, CA solubility drops below 40%, and excess undissociated CA molecules are dominated by hydrophobic interactions between phenolic rings, leading to the formation of insoluble aggregates and macroscopic precipitation, rather than participating in cross-linking between AP chains. Subsequent investigations were conducted with CA concentrations of 3–9 mg/mL, covering both the pre-gelation threshold state and stable gelation states of the AP–CA system.

### 3.2 Visual appearance and color difference of composite hydrogels

**Fig. 2:**
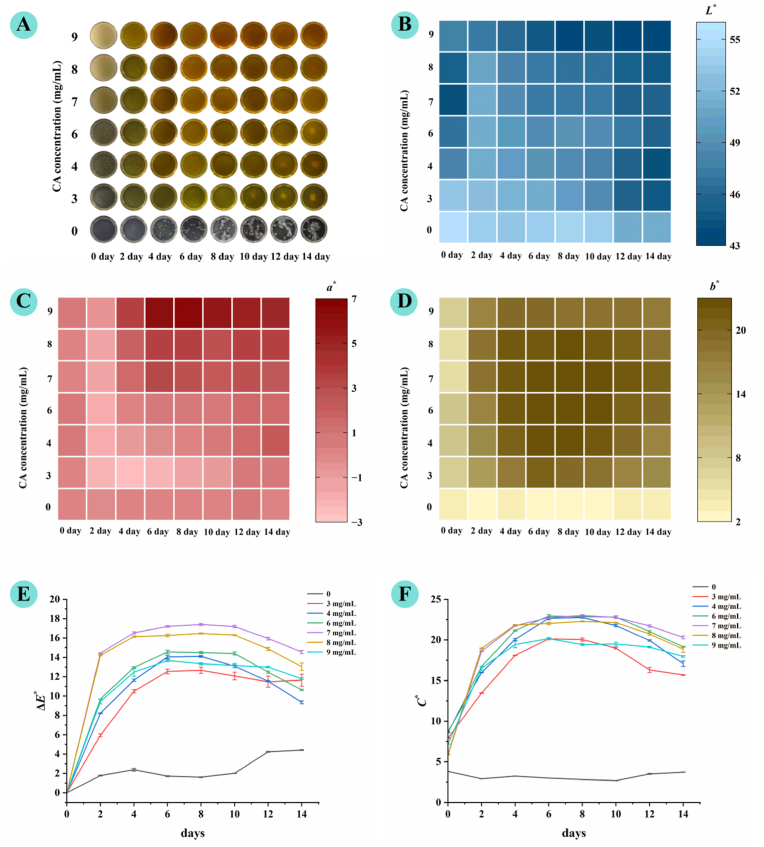
Appearance and color changes of AP–CA composite hydrogels at different CA concentrations during 14 days of observation. (**A**) Visual appearance of hydrogels photographed every 2 days in culture dishes; (**B**) Changes in *L*∗ values; (**C**) *a*∗ values; (**D**) *b*∗ values; (**E**) Δ*E*∗ values; (**F**) *C*∗ values.

Fig. 2 illustrates the visual appearance and color characteristics of AP dispersion and composite hydrogels containing different concentrations of CA during a 14-day storage period. Samples were placed in culture dishes, and their visual appearance and mold growth status were monitored every two days *via* visual observation. Color was quantified using the parameters *L*∗ (lightness), *a*∗ (red–green axis), and *b*∗ (yellow–blue axis). Representative images of all samples are shown in Fig. 2A, while Fig. 2B, C, and 2D present the corresponding heat maps of *L*∗, *a*∗, and *b*∗ values. The addition of CA markedly influenced the gel color, as reflected by reduced *L*∗ values and elevated *a*∗ and *b*∗ values, indicating that the gels became darker with a more pronounced red and yellow hue. Importantly, while the AP dispersion (control group) exhibited visible mold growth by day 3 *via* visual inspection, all CA-containing composite hydrogels showed no visually observable mold growth throughout the 14-day observation period, which indirectly indicates the bacteriostatic potential of CA within the AP-based hydrogel matrix. Due to microbial contamination, the color data of the control (AP dispersion) were considered unreliable beyond day 3. Over the storage period, the *a*∗ and *b*∗ values of the composite hydrogels showed a progressive increase, suggesting that the gels became redder and yellower compared to their freshly prepared counterparts. This progressive chromaticity shift was predominantly attributed to the autoxidation of CA: the phenolic hydroxyl groups of coumaric acid are susceptible to oxidative reactions during ambient storage, which generate colored quinone intermediates and subsequent low-molecular-weight oxidative derivatives, the primary contributors to the enhanced red and yellow chromaticity. Furthermore, as depicted in Fig. 2E and F, the chromaticity difference (Δ*E*∗) and chroma (*C*∗) values of the composite hydrogels exhibited a general trend of rising initially and then declining, with peak values observed between days 6 and 8.

### 3.3 Rheological properties of composite hydrogels

Rheological analysis was performed to characterize the viscoelastic behavior and network characteristics of composite hydrogels. The storage modulus (*G′*) reflects energy stored elastically, while the loss modulus (*G″*) reflects the energy dissipated viscously. Typically, *G′* > *G″* indicates a gel-like state, whereas *G′* < *G″* corresponds to a sol-like behavior (Liu et al., 2023).

#### 3.3.1 Strain sweep test

Dynamic strain sweep tests were first conducted to determine the linear viscoelastic region (LVR, defined as the strain range where *G′* maintains its plateau value with <10% reduction) and quantify the deformation resistance of AP dispersion and AP–CA composite hydrogels. As shown in Fig. S5, all composite hydrogels maintained a stable *G′* within the LVR, with *G′* consistently higher than *G″*, confirming the dominance of elastic behavior. Importantly, both *G′* and *G″* values were higher in AP–CA composite hydrogels compared to the control (AP dispersion), confirming that CA incorporation significantly enhanced viscoelasticity. Moreover, increasing CA concentration significantly elevated *G′* of composite hydrogels. For example, at the LVR, *G′* increased from 0.48 ± 0.02 Pa in the control to 13.92 ± 0.10 Pa in the composite hydrogel containing 9 mg/mL CA (Table 1). Concurrently, increasing CA concentration narrowed the LVR, which is a well-documented feature of rigid, highly crosslinked gel networks (Zhang et al., 2024).

**Table 1:** Approximate values of *G′* and *G″* in the linear viscoelastic region and the crossover strain of AP–CA composite hydrogels obtained from the strain sweep test.

AP–CA composite hydrogels with different CA concentrations	*G′* (Pa)	*G″* (Pa)	Crossover point strain (%)
3 mg/mL CA	0.48 ± 0.02a	0.23 ± 0.01a	ND
4 mg/mL CA	2.26 ± 0.02b	0.77 ± 0.01b	3.90 ± 0.01a
6 mg/mL CA	7.64 ± 0.05c	2.33 ± 0.12c	0.85 ± 0.00b
7 mg/mL CA	10.32 ± 0.03d	2.84 ± 0.10d	0.59 ± 0.01c
8 mg/mL CA	12.61 ± 0.10e	3.27 ± 0.11e	0.58 ± 0.01c
9 mg/mL CA	13.92 ± 0.10e	3.25 ± 0.15e	0.58 ± 0.00c

Note: Different letters in the same column indicate significant differences (*p* < 0.05). The control (AP dispersion) remained liquid state with no identifiable LVR, so its data are excluded.

Strain-induced viscoelastic transitions were further characterized by the crossover of *G′* and *G″*, which marks the yield point where the gel network collapses and viscous flow dominates. In the composite hydrogel containing 3 mg/mL CA, no crossover between *G′* and *G″* was detected, suggesting the absence of a viscoelastic transition (Fig. S5B, Table 1). In contrast, hydrogels with ≥4 mg/mL CA exhibited distinct crossover points, with critical strains decreasing from 3.90% ± 0.01% (4 mg/mL CA) to ∼0.58% (8–9 mg/mL CA) (Fig. S5C–G, Table 1). This downward shift in yield strain aligned with the narrowed LVR, confirming that higher CA crosslinking increased network rigidity but reduced tolerance to large deformation. Additionally, all hydrogels with ≥4 mg/mL CA exhibited weak strain overshoot behavior, characterized by a sharp *G′* decrease concurrent with a transient *G″* overshoot in the non-linear viscoelastic region (Kong et al., 2025). This phenomenon, typical of soft glassy materials, reflects the concurrent breakdown and reorganization of the three-dimensional gel network under deformation (Li et al., 2021). Comparable weak strain overshoot behavior has been reported in κ-carrageenan–carboxylated cellulose nanofiber–lychee polyphenol composite hydrogels (Qiu et al., 2025), further underscoring the role of polyphenol-mediated interactions in modulating the mechanical response of polysaccharide-based hydrogels.

#### 3.3.2 Frequency sweep test

**Fig. 3:**
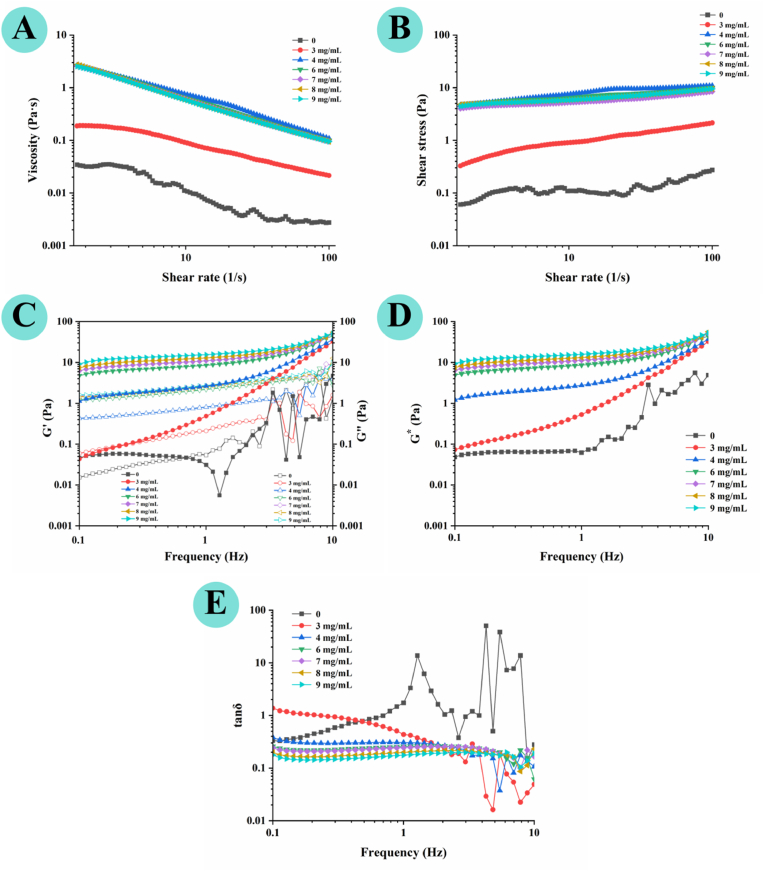
Rheological properties of AP–CA composite hydrogels. (**A**) apparent viscosity, (**B**) shear stress, (**C**) storage modulus (*G′*) and loss modulus (*G″*), (**D**) complex modulus (*G∗*), and (**E**) loss tangent (tan *δ*) under frequency sweep.

**Fig. 4:**
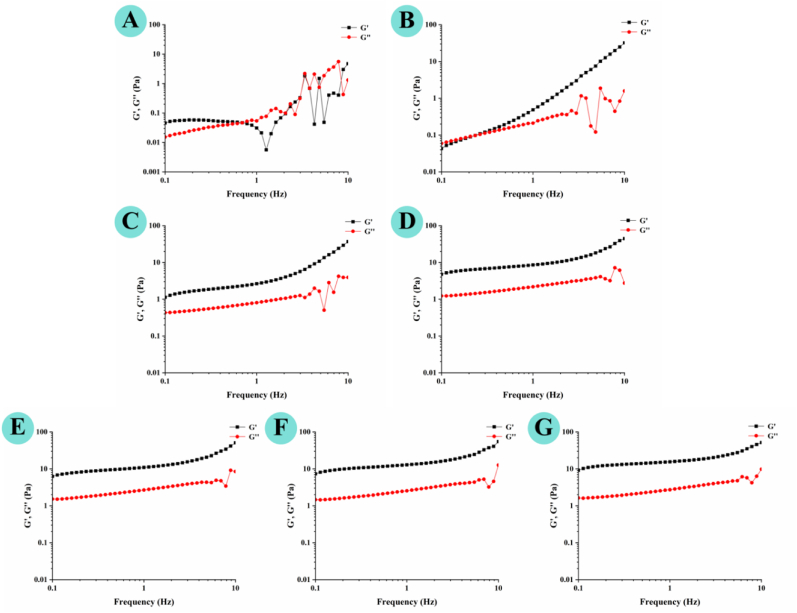
*G′* and *G″* of composite hydrogels at CA concentrations of 0, 3, 4, 6, 7, 8, and 9 mg/mL, respectively.

Frequency sweep tests were conducted within the LVR to evaluate the influence of frequency on the viscoelastic behavior of the hydrogels (Liu et al., 2023). As shown in Fig. 3, Fig. 4, AP dispersion exhibited low, frequency-fluctuating *G′* and *G″* values, reflecting weak, transient intermolecular forces. In contrast, CA incorporation markedly increased both *G′* and *G″* across the entire tested frequency range (0.1–10 Hz), with *G′* at 0.1 Hz increasing by over two orders of magnitude from 0.03 ± 0.01 Pa (AP dispersion) to 9.58 ± 0.35 Pa (9 mg/mL CA composite hydrogel). These marked enhancement confirms that CA acts as a crosslinker, promoting associations between AP chains to form a denser, more continuous network (Xie et al., 2025). For the 3 mg/mL CA hydrogel, *G″* exceeded *G′* at low frequencies, indicating viscous dominance, but *G′* surpassed *G″* at higher frequencies, suggesting a frequency-induced sol-gel transition (Fig. 4B). This behavior is typical of weakly crosslinked systems, where the network can relax and flow over long time scales but exhibits elastic response over short time scales. By contrast, composite hydrogels with ≥4 mg/mL CA maintained *G′* > *G″* across the frequency range with no crossover was observed, indicating an elastic-dominant and solid-like network (Fig. 4C–G). This transition from frequency-dependent fluid-like behavior to frequency-insensitive elastic behavior confirms the formation of a stable, highly crosslinked gel network at higher CA loadings.

The Power Law model was applied to quantitatively describe the frequency-dependent viscoelastic response and network relaxation characteristics, with fitting parameters summarized in Table 2. For the elastic modulus*G′* (*G′* = *K′ω*^*n*^*′*), the consistency index (*K′*) increased dramatically from 0.00 (AP dispersion) to 12.17 ± 0.39 Pa·s^n^ (9 mg/mL CA hydrogel), while the frequency-dependence exponent (*n′*) decreased from 2.54 ± 0.18 to 0.15 ± 0.01, indicating a shift from frequency-dependent, fluid-like behavior to frequency-insensitive elasticity as CA loading increases (Yang et al., 2018). Similar trends were found for the viscous modulus (*G″* = *K″ω*^*n*^*″*), with rising *K″* and decreasing *n″* values, suggesting suppressed viscous flow and restricted chain mobility in the crosslinked network. Identical evolution was found for the composite modulus (*G∗* = *A*_*α*_*ω*^*ᵅ*^), where the stiffness coefficient (*A*_*α*_) increased from 0.00 to 12.28 ± 0.34 and a decline in *α* (from 2.55 ± 0.73 to 0.15 ± 0.01) with increasing CA concentration. This evolution reflects enhanced rigidity and higher structural integrity, typical of well-developed gel networks (Zhong et al., 2024). Higher *G∗* values indicate greater total resistance to deformation, while lower *α* values imply reduced frequency dependence, consistent with the formation of stable elastic domains (Wang et al., 2022). The loss tangent (tan *δ* = *G″*/*G′*), an indicator of the viscoelastic balance, further supported this transition (Fig. 3E). All composite hydrogels with ≥4 mg/mL CA exhibited tan *δ* < 1 across the tested frequencies, and tan *δ* decreased progressively with increasing CA concentration, indicating enhanced elastic integrity and reduced internal energy dissipation of the network (Feng et al., 2023; Zhou et al., 2022).

**Table 2:** Parameters of power-law fitting describing storage modulus (*G′*), loss modulus (*G″*), material stiffness parameter (*A*_*α*_), and order of relaxation function (*α*) of AP–CA composite hydrogels.

Model	Parameters	Control (AP dispersion)	Composite gels (3 mg/mL CA)	Composite gels (4 mg/mL CA)	Composite gels (6 mg/mL CA)	Composite gels (7 mg/mL CA)	Composite gels (8 mg/mL CA)	Composite gels (9 mg/mL CA)
***G′ = K′w*^*n*^*′***	***K′***	0.00 ± 0.00a	0.01 ± 0.00a	0.06 ± 0.01a	5.79 ± 0.01b	7.46 ± 0.11c	8.98 ± 0.37d	12.17 ± 0.39e
	***n′***	2.54 ± 0.18d	1.97 ± 0.04c	1.56 ± 0.02b	0.22 ± 0.02a	0.21 ± 0.03a	0.18 ± 0.00a	0.15 ± 0.01a
	**R^2^**	0.99	0.99	0.99	0.99	0.99	0.99	0.99
***G″ = K″w*^*n*^*″***	***K″***	0.00 ± 0.00a	0.07 ± 0.00a	0.46 ± 0.01b	1.24 ± 0.01c	1.49 ± 0.08de	1.39 ± 0.07d	1.61 ± 0.00e
	***n″***	2.71 ± 0.02c	0.64 ± 0.00b	0.33 ± 0.01a	0.31 ± 0.01a	0.30 ± 0.00a	0.31 ± 0.00a	0.30 ± 0.00a
	**R^2^**	0.99	0.99	0.99	0.99	0.99	0.99	0.99
***G∗*=*A*_*α*_*w*^*α*^**	***A*_*α*_**	0.00 ± 0.00a	0.01 ± 0.00a	0.08 ± 0.01a	5.99 ± 0.06b	7.67 ± 0.25c	9.31 ± 0.15d	12.28 ± 0.34e
	***α***	2.55 ± 0.73c	1.98 ± 0.06bc	1.48 ± 0.03b	0.21 ± 0.00a	0.20 ± 0.00a	0.18 ± 0.01a	0.15 ± 0.01a
	**R^2^**	0.99	0.99	0.99	0.99	0.99	0.99	0.99

Note: Different lowercase letters indicate significant differences among different values in each row (*p* < 0.05).

Collectively, these results demonstrate that CA-mediated crosslinking drives the transition of the AP system from a weakly structured dispersion to a stable, elastic gel network with well-defined solid-like characteristics (Li et al., 2023).

#### 3.3.3 Apparent viscosity

Steady shear rheology was characterized to evaluate the flow behavior of AP–CA composite hydrogels, which is a critical parameter for their practical application in injectable delivery systems (Maiz-Fernández et al., 2021; Tong et al., 2024). As shown in Fig. 3A and B, the apparent viscosity of all samples decreased continuously with increasing shear rate, exhibiting typical pseudoplastic non-Newtonian behavior. The shear-thinning property arises from the shear-induced disruption of CA-mediated non-covalent interactions, reduced chain entanglement, and molecular alignment along the flow direction, which collectively lower flow resistance (Qiu et al., 2025; Tong et al., 2024). The Power Law model was applied to quantitatively describe the flow behavior of the hydrogels, with excellent fitting accuracy (R^2^ > 0.97) for all samples (Table 3). The consistency coefficient *K*, a measure of the material's intrinsic viscous resistance, increased from 0.09 ± 0.02 (AP dispersion) to 4.26 ± 0.10 Pa·s^n^ (4 mg/mL CA hydrogel), then remained relatively stable (3.57 to 4.11 Pa·s^n^) at higher CA loadings, consistent with the observed viscosity plateau. This trend suggests that crosslink density reaches a near-saturated state at 4 mg/mL CA, with additional CA having minimal effect on the steady-state structural resistance of the network under shear. The flow behavior index (*n*) was less than 1 for all samples, confirming the pseudoplastic characteristics of the hydrogels. Moreover, n values decreased progressively with higher CA concentrations, indicating more pronounced shear-thinning behavior at higher crosslink densities (Qiu et al., 2025).

**Table 3:** Power law parameters fitted from the apparent viscosity of AP–CA composite hydrogels at different CA concentrations.

AP–CA composite hydrogels with different CA concentrations	*K* (Pa·s^*n*^)	*n*	*R*^2^
Control (AP dispersion)	0.09 ± 0.02a	0.07 ± 0.08a	0.97
3 mg/mL CA	0.34 ± 0.01a	0.43 ± 0.03c	0.99
4 mg/mL CA	4.26 ± 0.10d	0.22 ± 0.02b	0.99
6 mg/mL CA	3.86 ± 0.06bc	0.23 ± 0.02b	0.99
7 mg/mL CA	3.57 ± 0.15b	0.23 ± 0.01b	0.99
8 mg/mL CA	4.11 ± 0.03cd	0.18 ± 0.00 ab	0.99
9 mg/mL CA	3.91 ± 0.11c	0.21 ± 0.01b	0.99

Note: Different letters in the same column indicate significant differences (*p* < 0.05).

Taken together, the steady shear rheology results demonstrate AP–CA composite hydrogels possess pronounced shear-thinning behavior, with tunable viscosity *via* CA loading. These properties are highly desirable for extrusion-based injectable applications, as the hydrogels can flow easily through a needle under high shear, then rapidly recover their elastic gel structure and mechanical strength after injection.

### 3.4 Texture profile analysis (TPA)

Texture is a critical quality attribute of food products, directly affecting consumer perception and acceptance. Texture Profile Analysis (TPA), commonly referred to as the “two-bite test”, is widely applied to simulate mastication and quantitatively characterize hydrogel texture (Matheus et al., 2024). The primary parameters include hardness, adhesiveness, springiness, and cohesiveness, while gumminess and chewiness are derived secondary parameters. Because the AP dispersion existed in liquid form, TPA measurements could only be performed on the AP–CA composite hydrogels. As summarized in Table 4, all composite hydrogels exhibited relatively high hardness and gumminess but low cohesiveness and resilience, indicating that the gels were brittle, prone to fracture under repeated compression, and had poor instantaneous energy recovery during rapid small deformation. This textural behavior resembles that reported for tea polyphenol-enhanced *Nicandra physalodes* seed polysaccharide gels (Ma et al., 2024). Gumminess and chewiness closely followed the trend of hardness, consistent with their dependence on gel strength. Cohesiveness, representing the internal bonding forces that prevent material from disintegration, ranged from 68% to 80% across samples, suggesting moderate structural integrity. In contrast, springiness values were notably high (93%-95%), indicating strong elastic recovery after deformation. Such high springiness can be attributed to the dense internal network structure, which enhances the structural integrity of the hydrogel system. However, the relatively low resilience values further confirm the brittle nature of these hydrogels, as they fractured easily under repeated compression. Adhesiveness, defined as the work required to detach the gel from a contacting surface, also emerged as a key determinant of gel stability during handling and storage. AP–CA composite hydrogels containing 6–9 mg/mL CA displayed high absolute adhesiveness values (238.65–249.08 g/s), which may enhance the storage stability of the hydrogel system. Interestingly, the hydrogel with 3 mg/mL CA exhibited comparatively higher springiness, resilience, and cohesiveness but lower hardness, indicating weaker gel strength yet greater ability to recover from deformation. Similar trends have been observed in curdlan-tannic acid hydrogel systems (Zhou et al., 2022).

**Table 4:** Texture parameters of AP–CA composite hydrogels.

AP–CA composite hydrogels with different CA concentrations	Hardness (g)	Fracturability (g)	Adhesiveness (g.sec)	Springiness	Cohesiveness	Gumminess	Chewiness	Resilience
3 mg/mL CA	218.84 ± 9.73b	220.35 ± 9.99cd	−109.23 ± 4.35c	0.94 ± 0.01a	0.80 ± 0.00c	145.77 ± 5.53a	143.64 ± 13.82a	0.30 ± 0.00b
4 mg/mL CA	227.88 ± 5.18b	227.05 ± 5.31cd	−194.96 ± 3.74b	0.94 ± 0.03a	0.68 ± 0.01a	154.55 ± 3.50a	145.29 ± 3.38a	0.09 ± 0.02a
6 mg/mL CA	237.00 ± 0.43c	235.82 ± 0.52d	−238.65 ± 8.78a	0.95 ± 0.01a	0.71 ± 0.02 ab	167.75 ± 5.58b	159.07 ± 3.69a	0.09 ± 0.04a
7 mg/mL CA	212.47 ± 8.67 ab	212.31 ± 8.93bc	−239.34 ± 12.98a	0.94 ± 0.01a	0.77 ± 0.04bc	167.92 ± 3.59b	152.89 ± 7.77a	0.12 ± 0.01a
8 mg/mL CA	199.83 ± 7.95a	198.57 ± 7.96 ab	−241.38 ± 13.07a	0.93 ± 0.01a	0.77 ± 0.03bc	154.12 ± 1.15a	142.79 ± 2.52a	0.14 ± 0.02a
9 mg/mL CA	197.56 ± 6.01a	196.54 ± 4.86a	−249.08 ± 2.94a	0.93 ± 0.01a	0.80 ± 0.06c	156.84 ± 7.98 ab	145.40 ± 9.65a	0.15 ± 0.04a

Note: Different letters in the same column indicate significant differences (*p* < 0.05). The control remained liquid state and thus could not undergo TPA, so its data are not included.

### 3.5 Turbidity and transparency

**Fig. 5:**
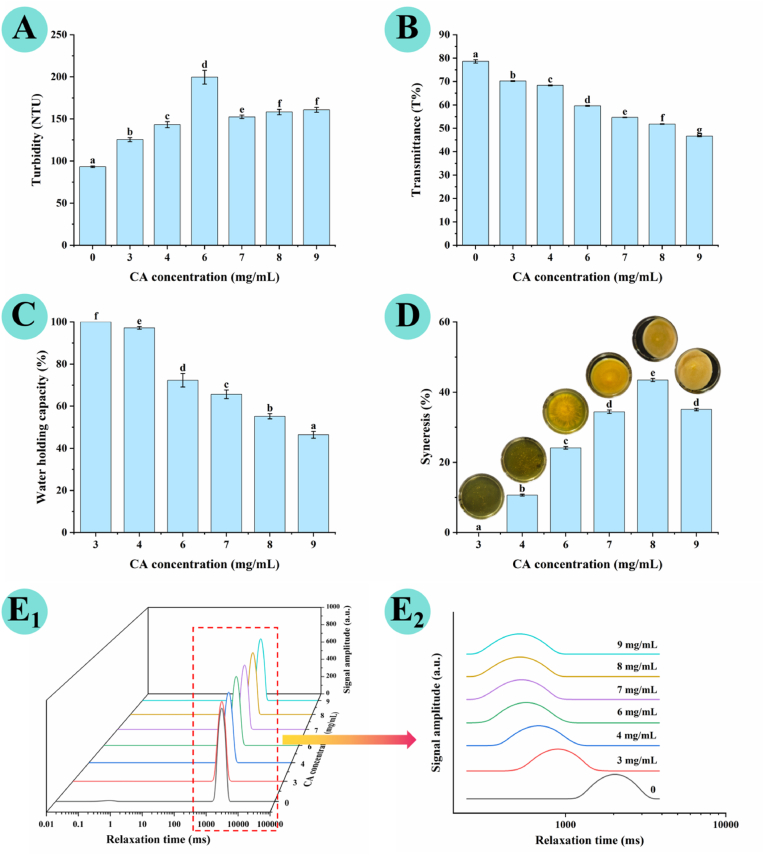
Characterization of AP–CA composite hydrogels. (**A**) turbidity, (**B**) transmittance, (**C**) water-holding capacity, (**D**) syneresis rate, (**E**) *T*_*2*_ relaxation time (**E_1_**: 3D waterfall plot, **E_2_**: 2D curve plot). Note: Different lowercase letters indicate significant differences (*p* < 0.05).

The turbidity and transmittance of AP dispersion and AP–CA composite hydrogels are shown in Fig. 5A and B. Incorporation of CA induced a dose-dependent reduction in gel transmittance, which decreased from 78.60% ± 0.71% in the control to 46.67% ± 0.21% in the composite hydrogel with 9 mg/mL CA. Correspondingly, the turbidity of AP–CA composite hydrogels rose progressively with increasing CA concentrations, peaking at 199.60 ± 8.21 NTU at 6 mg/mL CA, followed by a slight decrease at higher CA loadings (7–9 mg/mL). All composite hydrogels exhibited significantly higher turbidity than the control (93.23 ± 1.03 NTU) (Fig. 5A). This pronounced increase in turbidity provides strong evidence that CA promotes denser cross-linking within the composite gel matrix, thereby significantly modifying its optical properties. These changes in optical properties are closely associated with increased microstructural heterogeneity in the system after CA incorporation. CA forms extensive non-covalent interactions with AP chains, which drive the formation of intermolecular aggregates and crosslinked network domains. These inhomogeneous microstructures enhance the scattering of incident light, which macroscopically manifests as elevated turbidity and reduced transmittance (Fang et al., 2025). Similar phenomena have been widely reported in polyphenol-modified biopolymer systems. For instance, Wang et al. (2024) observed increased opacity in tea polyphenol-reinforced egg white protein gels, and Qiu et al. (2025) reported a marked reduction in transparency of litchi polyphenol-modified κ-carrageenan–carboxylated cellulose nanofiber composite hydrogels. Both studies attributed the optical changes to polyphenol-mediated intermolecular interactions that alter the microstructural uniformity of the matrix.

### 3.6 Syringeability of hydrogels

Syringeability, defined as the ability of a hydrogel to be smoothly extruded from a syringe while retaining a predefined structure, is an essential requirement for injectable biomedical therapies and bioink applications (Kong et al., 2025; You et al., 2022). Rheological analysis confirmed that AP–CA composite hydrogels exhibit pronounced shear-thinning behavior, which enables facile extrusion through a needle, and rapid recovery of viscoelastic properties upon shear stress removal, which is essential for maintaining structural fidelity after injection (Liu et al., 2024). In addition, the storage modulus (*G′*, a key indicator of mechanical strength and network stability) increased with CA concentration, suggesting that CA incorporation enhances the hydrogel's shape retention capacity after extrusion (Ettoumi et al., 2024).

**Fig. 6:**
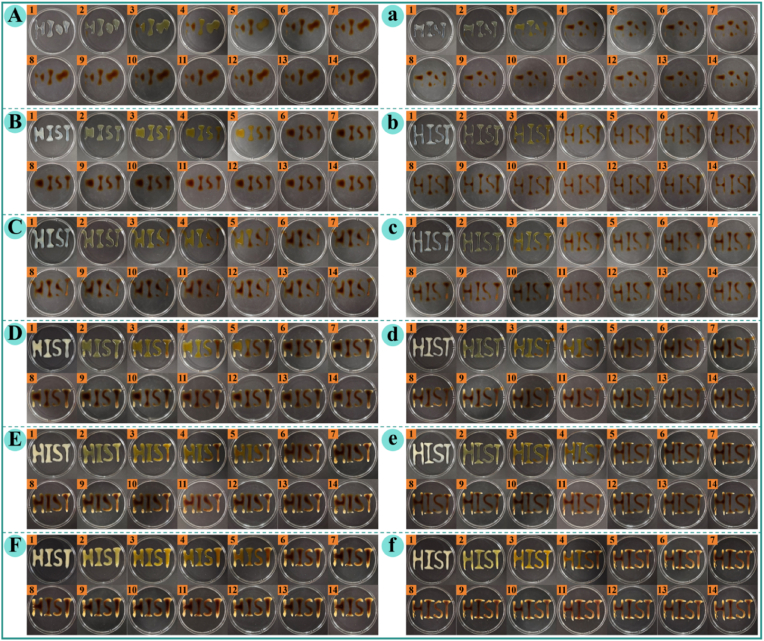
Syringeability of AP–CA composite hydrogels at different CA concentrations during 14 days of observation. (**A–F**) Hydrogels injected with a coarse syringe (5 mL), and (**a–f**) hydrogels injected with a fine syringe (1 mL), corresponding to CA concentrations of 3, 4, 6, 7, 8, and 9 mg/mL, respectively. Note: Numbers 1–14 in the upper left corner of each sub-panel indicate the corresponding storage days after extrusion.

The syringeability and long-term structural stability of the hydrogels were visually evaluated by extruding a predefined “HIST” pattern using both coarse (5 mL) and fine (1 mL) syringes (Fig. 6). **Panels A–F** show hydrogels extruded with the coarse syringe, whereas **panels a–f** depict those from the fine syringe, corresponding to composite hydrogels with CA concentrations of 0, 3, 4, 6, 7, 8, and 9 mg/mL, respectively. The numbers 1–14 in the upper left corner of each panel indicate the storage days after extrusion. AP dispersion failed to maintain any structural integrity, collapsing into irregular pools immediately after extrusion, confirming its lack of self-supporting ability. In contrast, all AP–CA composite hydrogels were extruded smoothly without nozzle blockage, demonstrating favorable flowability under shear. For the 3 mg/mL CA hydrogel, the extruded hydrogel had poor structure definition: the coarse syringe produced a distorted “S” character, with structural merging occurred by day 3, while the fine syringe yielded discontinuous, broken patterns. The 4 mg/mL CA hydrogel produced a more recognizable “HIST” structure, although partial blurring and aggregation of the “H” character occurred after 2 days of storage. Remarkably, composite hydrogels containing 6–9 mg/mL CA produced well-defined, continuous “HIST” patterns, which remained intact with no obvious distortion or merging for at least 14 days of storage.

The superior syringeability and shape retention of AP–CA composite hydrogels can be attributed to their dynamic shear-thinning rheology. Under extrusion shear stress, reversible interactions temporarily dissociate, reducing flow resistance and enabling smooth extrusion. Upon stress removal, the interactions rapidly reassemble, restoring gel strength and preserving the extruded architecture (Ettoumi et al., 2024). At low CA concentrations (3 and 4 mg/mL), the low *G′* and weak mechanical strength cannot support long-term structural stability, leading to pattern distortion and merging. Conversely, higher CA concentrations (6–9 mg/mL) enhanced intermolecular associations, resulting in a more robust elastic network with improved viscoelastic strength and dimensional stability, which ensures long-term structural fidelity of the injectable constructs.

### 3.7 Water holding capacity (WHC) and syneresis rate (SR) evaluation

WHC and SR are two complementary critical indicators reflecting the water retention performance of hydrogels: WHC characterizes the water entrapment efficiency and structural integrity of the gel network under strong centrifugal mechanical stress, while SR reflects freeze–thaw stability *via* water expulsion behavior after freeze–thaw cycling (Qiu et al., 2025). Higher WHC and lower SR collectively represent superior water retention capacity and structural robustness of the hydrogel system, and their variation trends in this study are highly consistent, both revealing the regulation effect of CA concentration on the structural integrity and damage resistance of AP–CA composite hydrogels (rKazemi-Taskooh and Varidi, 2021).

As shown in Fig. 5C, the composite hydrogel with 3 mg/mL CA exhibited the highest WHC. This excellent water retention performance is directly supported by microstructure characterization results (Fig. 10, Table S2): the 3 mg/mL CA group had a high vessel percentage area (35.13% ± 2.46%), high total number of junctions (527.67 ± 82.04), and low mean lacunarity (0.15 ± 0.02), indicating the formation of a homogeneous, highly interconnected elastic network with uniform pore distribution. This continuous and robust skeleton structure can effectively maintain structural integrity under high centrifugal force (10,000 × g), and firmly immobilize water molecules trapped in the network pores, thus achieving the highest WHC. However, WHC decreased gradually with further increases in CA concentration. This phenomenon can be attributed to the dual effects of enhanced intermolecular association at high CA loadings, with direct evidence from microstructure analysis: on one hand, stronger interactions between AP and CA at high CA concentrations lead to a denser network structure, which restricts the mobility of water molecules under static conditions (consistent with LF-NMR results) (Hazrati and Madadlou, 2021); on the other hand, excessive cross-linking induces significant structural inhomogeneity (Gong et al., 2022; Karppanen et al., 2023; Zhu et al., 2024). As shown in Table S2, when CA concentration exceeded 6 mg/mL, the vessel percentage area and total number of junctions of the hydrogels decreased significantly, while mean lacunarity increased markedly, and SEM images showed enlarged irregular pores, thickened pore walls and locally collapsed frameworks (Fig. 10). This over-associated heterogeneous network has significantly increased rigidity and brittleness, with abundant local mechanical defects in the skeleton. Under high centrifugal stress, the brittle network is prone to irreversible local collapse at the defect sites, resulting in the release of water originally trapped in the network pores, thus manifesting as reduced WHC. Similar trends have been reported in other polyphenol–polysaccharide hydrogel systems, such as gellan gum-gallic acid composite hydrogels (Xu et al., 2024).

Syneresis occurs when ice crystal formation during freezing disrupts the hydrogel network structure, leading to phase separation and water release upon thawing. with lower SR indicating stronger resistance to freeze–thaw damage (Srichuwong et al., 2012). As shown in Fig. 5D, the composite hydrogel with 3 mg/mL CA exhibited the lowest SR, further confirming its enhanced stability under freeze–thaw conditions. The variation trend of SR is also fully supported by microstructure characterization: the uniform, highly interconnected elastic network formed at 3 mg/mL CA can effectively buffer the expansion stress generated by ice crystal growth during freezing, avoiding irreversible structural damage, thus minimizing water expulsion after thawing. In contrast, the brittle and heterogeneous network formed at high CA concentrations (≥6 mg/mL) has poor resistance to ice crystal-induced damage. Ice crystals preferentially grow and expand in the weak areas of the network, causing irreversible tearing of the gel skeleton and phase separation, which eventually leads to a significant increase in SR. The 8 mg/mL CA group, which had the lowest vessel percentage area (30.08% ± 3.21%) and the highest lacunarity (0.19 ± 0.03) among all composite hydrogels, showed the most severe structural inhomogeneity and thus the highest SR (43.45% ± 0.45%). These findings suggest that AP–CA composite hydrogels have tunable water retention and freeze–thaw stability *via* CA concentration regulation, which endows them with application potential in frozen food systems to prevent moisture loss and texture deterioration during cold storage (Shang et al., 2021; Tang et al., 2021).

### 3.8 LF-NMR analysis

Water plays a critical role in hydrogel systems, and its mobility and distribution under static, non-destructive conditions provide insights into the compactness of the hydrogel matrix, which complements the mechanical stress-related water retention performance characterized by WHC. In this study, LF-NMR was employed to evaluate the water dynamics of AP dispersion and AP–CA composite hydrogels. The spin–spin relaxation time (*T*_*2*_) is inversely proportional to water mobility: shorter *T*_*2*_ values indicate stronger restriction of water motion by the polymeric network (Hu and Meng, 2025). The *T*_*2*_ distribution curves obtained by multi-exponential fitting showed three characteristic relaxation peaks in the range of 0.01–10,000 ms: *T*_*21*_ (0.01–10 ms, bound water), *T*_*22*_ (10–100 ms, semi-bound water), and *T*_*23*_ (100–1000 ms, free water) (Kopač et al., 2022; Zhong et al., 2024).

As shown in Fig. 5E, all hydrogel samples exhibited a dominant relaxation peak assigned to *T*_*23*_, with the relative peak area exceeding 99% in all groups, confirming that free water is the main form of water in the AP–CA hydrogel system. Notably, with increasing CA concentration, the *T*_*23*_ peak shifted gradually toward shorter relaxation times, with a simultaneous decrease in peak intensity. The *T*_*23*_ value decreased from 2009.23 ms for AP dispersion to 546.23–890.22 ms for the AP–CA composite hydrogels. This pronounced decline in *T*_*23*_ indicated that the intermolecular association between AP and CA is enhanced with increasing CA concentration, forming a denser three-dimensional network that imposes stronger physical restriction on the motion of free water molecules under static state (Xu et al., 2024). The progressive decline in *T*_*2*_ is consistent with the increasing trend of *G′* and *G″* in rheological analysis, both of which reflect the strengthened intermolecular interactions and tighter network architecture induced by CA incorporation (Kopač et al., 2022).

It is important to note that the *T*_*2*_ value from LF-NMR and WHC reflect two distinct performance dimensions of the hydrogel, with no inherent contradiction between the two sets of results. LF-NMR characterizes the mobility of water molecules in the hydrogel under static, force-free conditions, which is determined by the compactness of the network structure; while WHC reflects the water retention capacity of the hydrogel under strong centrifugal mechanical stress, which is dominated by the structural integrity, connectivity and and brittleness resistance of the network under external force. The continuous decrease in *T*_*2*_ with increasing CA concentration confirms the persistent densification of the network and enhanced restriction of static water motion, while the decrease in WHC at high CA loadings is caused by the increased brittleness, reduced connectivity and centrifugation-induced structural collapse of the network, which is fully supported by SEM and AngioTool quantitative results.

### 3.9 Antioxidant, antibacterial, and probiotic activities

Antioxidant and antimicrobial properties are critical functional attributes for hydrogels applied in food preservation and biomedical fields, as they can mitigate oxidative degradation and microbial spoilage of the matrix. In this section, the antioxidant, antibacterial, and probiotic compatibility of AP–CA composite hydrogels were systematically characterized, with the individual contributions of AP and CA to each functional property clearly distinguished.

**Fig. 7:**
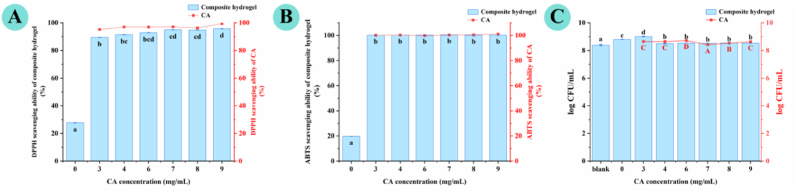
Antioxidant activities, including (**A**) DPPH and (**B**) ABTS radical scavenging assays, and (**C**) probiotic properties of AP–CA composite hydrogels at different CA concentrations. Note: Different lowercase letters indicate significant differences (*p* < 0.05).

The antioxidant activities of AP dispersion, CA solution, and AP–CA composite hydrogels were assessed using DPPH and ABTS radical scavenging assays, with results shown in Fig. 7A and B. AP dispersion exhibited weak but detectable radical scavenging capacity (DPPH: 27.7% ± 3.4%; ABTS: 19.7% ± 1.1%), which is attributed to the hydrogen-donating groups (*e.g*., uronic acids, hydroxyl groups) in the pectin backbone, representing the intrinsic antioxidant contribution of the AP matrix (Liu et al., 2023, 2024). In contrast, CA solution displayed potent antioxidant activity against both DPPH and ABTS radicals, confirming that phenolic hydroxyl groups of CA are the dominant source of antioxidant activity in the system. Incorporation of CA into the AP matrix significantly enhanced radical scavenging ability of the composite hydrogels in a CA concentration-dependent manner. At 3 mg/mL CA loading, the DPPH scavenging rate of the composite hydrogel approached ∼90%, while the ABTS scavenging rate exceeded 95%, which was comparable to the activity of the corresponding CA solution alone. Notably, radical scavenging activity plateaued at CA concentrations above 3 mg/mL: DPPH scavenging rate increased only marginally from 89.43% to 95.73% as CA rose from 3 to 9 mg/mL, while ABTS scavenging remained nearly constant above 99%. This trend is driven by two key factors: (i) reaction saturation, as 3 mg/mL CA provides sufficient phenolic hydroxyl hydrogen donors to scavenge nearly all available radicals; (ii) reduced radical accessibility, where enhanced AP–CA non-covalent interactions at high CA loadings partially encapsulate CA's active sites within the dense gel network, limiting access to lipophilic DPPH radicals, while the hydrophilic AP matrix maintains favorable contact for water-soluble ABTS radicals (Ma et al., 2024).

**Fig. 8:**
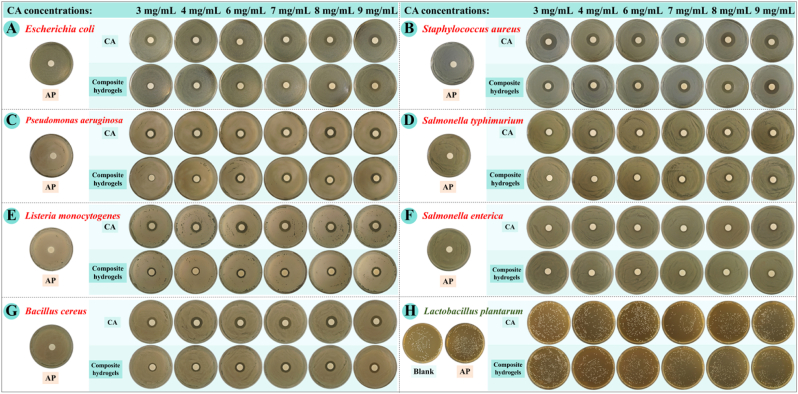
Antibacterial and probiotic properties of AP–CA composite hydrogels. (**A–G**) *In vitro* antibacterial activity of hydrogels against *Escherichia coli*, *Staphylococcus aureus*, *Pseudomonas aeruginosa, Salmonella typhimurium, Listeria monocytogenes*, *Salmonella enterica,* and *Bacillus cereus*; (**H**) Growth-promoting effect on *Lactobacillus plantarum*.

**Fig. 9:**
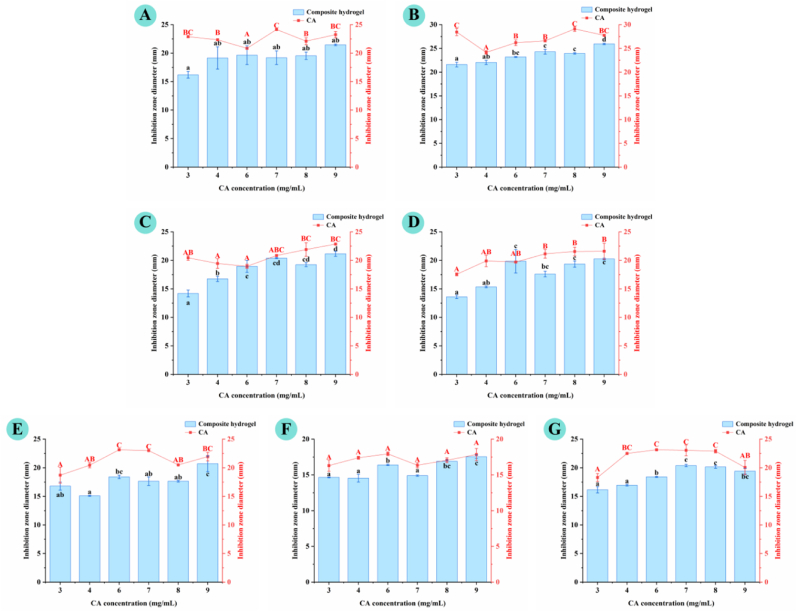
Inhibition zone diameter of AP–CA composite hydrogels against (**A**) *Escherichia coli*, (**B**) *Staphylococcus aureus*, (**C**) *Pseudomonas aeruginosa,* (**D**) *Salmonella typhimurium,* (**E**) *Salmonella enterica,* (**F**) *Bacillus cereus*, and (**G**) *Listeria monocytogenes*. Note: Different letters indicate significant differences (*p* < 0.05).

The antibacterial properties of the samples were evaluated against seven common foodborne and pathogenic bacterial strains, including four Gram-negative strains (*Escherichia coli*, *Pseudomonas aeruginosa*, *Salmonella typhimurium*, *Salmonella enterica*) and three Gram-positive strains (*Staphylococcus aureus*, *Bacillus cereus*, *Listeria monocytogenes*). Antibacterial activity was qualitatively visualized *via* inhibition zone assays (Fig. 8A–G) and quantitatively determined by inhibition zone diameter (Fig. 9). AP dispersion exhibited negligible antibacterial activity against all tested strains, with no observable inhibition zones, confirming that AP itself has no bacteriostatic effect. In contrast, CA solution showed clear antibacterial efficacy across all strains, demonstrating that CA is the core antibacterial functional component of the composite system. AP–CA composite hydrogels produced distinct inhibition zones against all tested strains, with inhibition zone diameters increasing alongside CA concentrations. For instance, the inhibition zone diameter ranged from 16.20 ± 0.60 to 21.45 ± 0.15 mm for *E. coli*, 21.60 ± 0.50 to 25.95 ± 0.15 mm for *S. aureus*, and 15.10 ± 0.10 to 20.70 ± 1.40 mm for *L. monocytogenes*. Notably, the inhibition zone diameters of the composite hydrogels were slightly lower than those of the corresponding CA solutions at the same CA loading. This difference is attributed to the diffusion behavior of CA, rather than a reduction in its intrinsic antibacterial activity, which directly addresses the question of gel encapsulation's effect on CA performance. CA solution can diffuse freely and instantaneously in the agar medium, forming a rapid high local concentration within the incubation period, resulting in a larger inhibition zone. In contrast, CA encapsulated in the hydrogel network is released in a sustained and gradual manner *via* diffusion from the gel matrix, leading to a lower instantaneous local concentration in the medium within the same incubation window, thus presenting a slightly smaller inhibition zone.

Pectin has been widely reported to have prebiotic properties by supporting the growth of beneficial probiotic strains (Derebasi et al., 2024). The compatibility of AP–CA composite hydrogels with *Lactobacillus plantarum* was evaluated. As shown in Figs. 7C and 8H, compared with the blank control (normal saline), the AP dispersion showed a significant increase in the viable count (log CFU/mL) of *L. plantarum*, confirming the inherent growth-promoting effect of AP as a prebiotic substrate for probiotic metabolism. For AP–CA composite hydrogels, a concentration-dependent effect on probiotic growth was observed: composite hydrogels with low CA concentrations (3–4 mg/mL) further enhanced the viable count of *L. plantarum*, with the highest count observed at 3 mg/mL CA loading. With the increase of CA concentration to 6 mg/mL and above, the growth-promoting effect decreased gradually, but the viable count of *L. plantarum* remained significantly higher than the blank control (normal saline), and was comparable to the pure AP group. Notably, even at the highest tested CA concentration of 9 mg/mL, no significant growth inhibition of *L. plantarum* was observed after incubation, confirming the good *in vitro* biocompatibility of the composite hydrogel system with the tested probiotic strain over the full CA concentration range.

The balance between the bioactivity of CA and the viability of *L. plantarum* was further interpreted based on the experimental results. On one hand, CA exhibits selective antibacterial activity: it exerts a broad-spectrum and potent inhibitory effect on the tested foodborne pathogenic bacteria, while showing no adverse effect on *L. plantarum* growth within incubation even at high concentrations. On the other hand, the AP matrix provides a favorable growth environment for the probiotic *via* its prebiotic activity, while the three-dimensional network structure of the hydrogel enables sustained release of CA, avoiding instantaneous high concentrations of free CA that may cause potential stress to probiotics. The synergistic effect between AP's prebiotic activity and CA's antioxidant/antibacterial activity gives the composite hydrogel excellent functional properties and good probiotic compatibility across a wide CA concentration range, indicating its potential as a probiotic delivery carrier. It should be noted that this study only evaluated the *in vitro* growth-promoting effect of the composite hydrogels on *L. plantarum*, and did not conduct simulated gastrointestinal digestion experiments to assess probiotic survival under gastric acid and bile salt stress, nor did it evaluate the long-term storage stability of probiotics in the hydrogel system. In follow-up studies, simulated gastrointestinal release experiments, long-term storage stability tests, and *in vivo* probiotic viability evaluation will be systematically conducted to fully verify the protective encapsulation effect of the AP–CA composite hydrogel system, and to clarify its application potential in probiotic delivery.

### 3.10 Microstructure

**Fig. 10:**
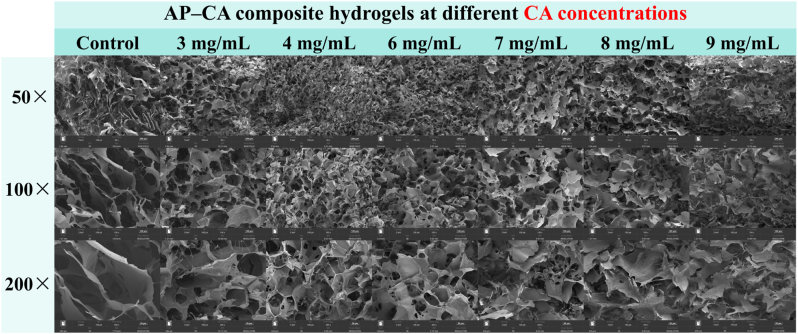
SEM micrographs of AP–CA composite hydrogels at different CA concentrations.

The microstructural features of the composite hydrogels were examined using scanning electron microscopy (SEM) combined with quantitative analysis *via* AngioTool software (Fig. 10, Fig. S6). Three parameters including vessel percentage area, total number of junctions, and mean lacunarity were used to characterize pore abundance, network connectivity, and structural uniformity, respectively (Table S2). Dried AP showed a disordered and loosely packed structure composed of irregular lamellar fragments and large, non-uniform voids. These observations were consistent with its low vessel area (24.66% ± 2.57%) and junction number (289.50 ± 82.63), together with the highest lacunarity (0.27 ± 0.06), indicating a weakly connected, poorly organized network. Incorporation of CA markedly reorganized the internal architecture of the hydrogels. At 3, 4 and 6 mg/mL CA, the hydrogels developed into a compact, continuous honeycomb-like network characterized by uniformly distributed micrometer-sized pores (Zhang et al., 2024). Correspondingly, AngioTool analysis showed substantial increases in vessel percentage area and junction density, along with pronounced reductions in lacunarity. The 4 mg/mL CA hydrogel exhibited the most favorable structural features, with the highest pore abundance (42.27% ± 1.99%) and network connectivity (704.33 ± 66.01) and the lowest lacunarity (0.11 ± 0.01), indicating a well-developed and highly interconnected polymer network. At higher CA concentrations (7–9 mg/mL), the structure became increasingly heterogeneous. Both vessel area and junction number declined (*e.g.*, 30.08% ± 3.21% at 8 mg/mL), while lacunarity increased (0.17–0.19). SEM images revealed enlarged, irregular pores and partially collapsed frameworks with thickened walls. This structural deterioration likely resulted from excessive hydrogen bonding and hydrophobic association between CA and AP: while moderate CA levels facilitate uniform crosslinking and stabilize the network, excessive CA induces local over-association and phase separation, leading to non-uniform pore enlargement, reduced network connectivity and increased structural brittleness (Yan et al., 2021). This pattern aligns with the rheological behavior of the hydrogels, in which moderate CA concentrations enhanced *G′*, while higher levels produced limited additional strengthening or even a slight decline (Zhang et al., 2024). More importantly, the microstructure evolution is fully consistent with the water retention performance of the hydrogels: the homogeneous, highly interconnected network formed at moderate CA concentrations (3–4 mg/mL) confers excellent structural integrity, leading to the highest WHC and lowest SR; while the heterogeneous, brittle network formed at excessive CA concentrations directly results in the deterioration of water retention and freeze–thaw stability.

Overall, SEM visualization coupled with AngioTool quantification reveals a clear concentration-dependent mechanism underpinning CA-induced spontaneous gelation. Moderate CA concentrations generate fine, uniform, and highly interconnected porous structures with low lacunarity, conferring enhanced mechanical integrity, water retention capacity, and freeze–thaw stability. In contrast, both insufficient and excessive CA result in suboptimal architectures—either too loose or overly aggregated and brittle—ultimately compromising gel performance. These results highlight the importance of balancing non-covalent intermolecular interactions and structural homogeneity in designing polyphenol-crosslinked biopolymer hydrogels.

### 3.11 Thermogravimetric (TG) and derivative thermogravimetry (DTG) analysis

**Fig. 11:**
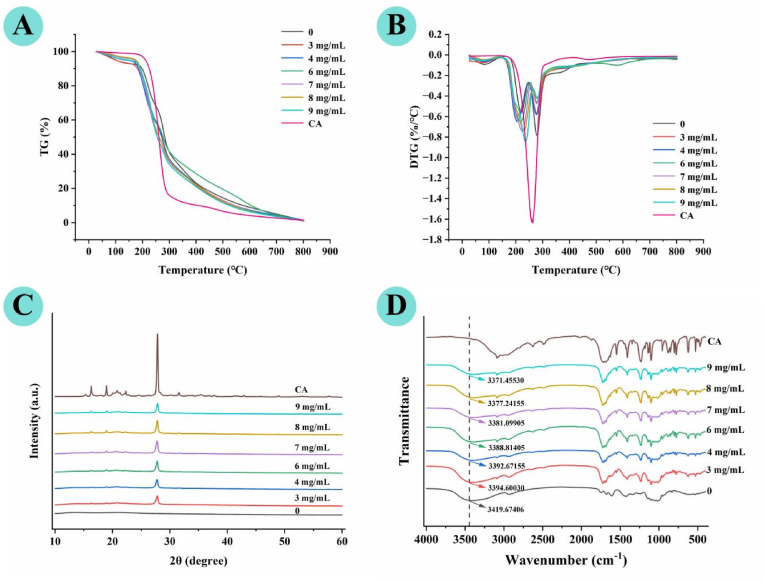
Structural characterization of AP–CA composite hydrogels: (**A**) TG thermograms, (**B**) DTG thermograms, (**C**) X-ray diffraction patterns, and (**D**) FT-IR spectra.

The TG and DTG curves of dried AP, CA, and AP–CA composite hydrogels are presented in Fig. 11A and B. All samples exhibited three distinct stages of thermal degradation. The first stage (30–150 °C) corresponded to a slight mass loss of approximately 15%, which is mainly attributed to the evaporation of adsorbed and weakly bound water within the hydrogel matrix. The second stage (150–270 °C) was the major decomposition region, with a rapid weight loss of about 60%. This phase primarily involved the cleavage of the pectin backbone, ring opening of sugar residues, decarboxylation reactions, and scission of C–C bonds within the pyranose rings, as well as the thermal decomposition of CA. Within this temperature range, consistent trends in thermal decomposition peaks were observed across samples. The pyrolysis peak of AP appeared at 228.6 °C, while that of the composite hydrogels showed an initial decrease and followed by an increase with rising CA concentration. Specifically, the peak temperatures declined to 206.50 °C and 204.99 °C at CA concentrations of 3 and 4 mg/mL, respectively, but increased to 230.05 °C and 236.57 °C at 8 and 9 mg/mL, respectively. All peak temperatures of composite hydrogels remained lower than that of pure CA (263.27 °C). These results suggest that CA incorporation alters the thermal degradation pathway of the AP matrix. At low CA concentrations, the introduction of CA may form locally heterogeneous regions in the AP matrix, leading to a slight reduction in thermal stability. At higher CA concentrations, theenhanced thermal resistance is consistent with the formation of stronger intermolecular associations between AP and CA, which restrict the thermal motion of polymer chains and improve the structural integrity of the matrix. In the third stage (270–350 °C), the degradation rate gradually decreased, and residual mass approached a plateau. With increasing CA concentration, the DTG peaks of composite hydrogels became broader and shallower, with a shift toward higher temperatures. This was accompanied by a reduction in the maximum weight-loss rate and a progressive rise in the corresponding peak temperature (Tong et al., 2024). Collectively, these thermal degradation characteristics indicate that the thermal stability of AP–CA composite hydrogels is regulated by CA content, with high CA loading endowing the hydrogel matrix with improved resistance to thermal degradation (Zhang et al., 2024).

### 3.12 X-ray diffraction (XRD) and FT-IR analysis

The XRD patterns of dried AP, CA, and AP–CA composite hydrogels are presented in Fig. 11C, with all patterns recorded in the 2θ range of 10°–60°. Crystallinity percentages were calculated using Jade software, based on the integrated area ratio of crystalline regions to total (crystalline + amorphous) regions. Pure AP exhibited a broad, featureless diffraction profile with no sharp characteristic peaks, confirming its predominantly amorphous nature. In contrast, CA displayed multiple sharp, intense peaks with a dominant characteristic peak at 27.8°, corresponding to its pronounced crystallinity (85.25%). For AP–CA composite hydrogels, the characteristic diffraction peaks of CA were retained, but their relative intensities were significantly reduced compared with pure CA, suggesting the disruption of CA crystalline domains after incorporation into the AP matrix (Vityazev et al., 2020).

FT-IR spectroscopy was employed to characterize the chemical structure and potential intermolecular interactions within the AP–CA composite hydrogels (Yang et al., 2025). The spectra of pure AP, pure CA, and AP–CA composite hydrogels are presented in Fig. 11D. In the spectrum of CA, a distinct stretching vibration of carbonyl (–C=O) was observed at ∼1715 cm^−1^, accompanied by a characteristic benzene ring stretching peak at ∼1615 cm^−1^. For AP, a broad and intense band at 3412 cm^−1^ was attributed to O–H stretching vibrations of hydroxyl groups in the pectin backbone. Additional characteristic peaks were observed at 2930, 1636, 1418, and 1026 cm^−1^, corresponding to C–H stretching (CH, CH_2_, CH_3_), asymmetric COO^−^ stretching, symmetric COO^−^ stretching, and C–O–C vibrations, respectively. The glycosidic bond of galacturonic acid contributed a symmetric C–O–C stretching vibration at 1250 cm^−1^, while the absorption peak at 1148 cm^−1^ was assigned to C–O stretching within the polysaccharide ring. In the spectra of AP–CA composite hydrogels, the characteristic peaks of both AP and CA were retained, confirming the successful incorporation of CA into the AP matrix. The –OH stretching peak of AP shifted toward lower wavenumbers, suggesting the formation of intermolecular hydrogen bonding between the hydroxyl/carboxyl groups of AP and the phenolic hydroxyl groups of CA. Notably, no crystalline CA was detected, confirming that CA was molecularly integrated into the polymer matrix (Chen et al., 2024). These concentration-dependent structural trends are consistent with FT-IR results, where red-shifts in O–H and C=O stretching bands reflect strengthened hydrogen bonding between CA and AP (Zhou et al., 2022). Such hydrogen bonding interactions may contribute to the cross-linking of the two components and the stabilization of the three-dimensional hydrogel network, which aligns with previously reported quaternized chitin–tannic acid hydrogel systems (Xie et al., 2025).

### 3.13 Intermolecular forces of composite hydrogels

**Fig. 12:**
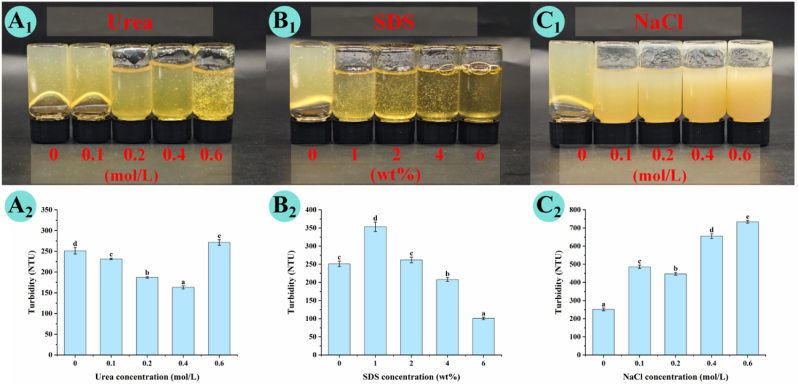
Changes in appearance (subscript 1) and turbidity (subscript 2) of AP–CA composite hydrogels after treatment with (**A**) urea, (**B**) SDS, and (**C**) NaCl. Note: Different lowercase letters indicate significant differences (*p* < 0.05). Prior to testing, optimization steps for degassing were performed.

The formation and stabilization of polysaccharide-based hydrogels are primarily governed by non-covalent interactions, among which hydrogen bonding, hydrophobic interactions, and electrostatic effects play critical roles. Urea, SDS, and NaCl, are widely employed as specific molecular probes to characterize these interactions: urea disrupts intermolecular hydrogen bonds by competing for hydrogen bond donor/acceptor sites, SDS interferes with hydrophobic interactions *via* its lipophilic alkyl chain, and NaCl modulates electrostatic effects by altering system ionic strength and shielding long-range electrostatic interactions between charged polymer chains (Feng et al., 2023). In this study, the intermolecular forces driving AP–CA composite hydrogel formation were elucidated by evaluating changes in hydrogel appearance and turbidity after treatment with gradient concentrations of urea, SDS, and NaCl, with the representative AP–CA hydrogel containing 6 mg/mL CA selected fo analysis (Fig. 12).

Urea treatment (Fig. 12A_1_-A_2_) induced complete loss of hydrogel self-supporting capacity and a significant turbidity decrease, confirming hydrogen bonding as the dominant driving force for hydrogel stabilization. The abnormal turbidity increase at 0.6 M urea was attributed to bubble interference during sample treatment, rather than matrix structural changes. SDS treatment (Fig. 12B_1_-B_2_) caused gradual hydrogel destabilization and continuous turbidity reduction, with a weaker disruptive effect than urea, indicating hydrophobic interactions serve as an auxiliary stabilizing force. NaCl treatment (Fig. 12C_1_-C_2_) resulted in loss of hydrogel self-supporting structure and continuous turbidity increase. Given both AP and CA carry negative charges at the experimental pH (3.0), intrinsic interchain electrostatic repulsion would inherently inhibit chain aggregation and gelation. This result supports the inference that CA incorporation mediates partial shielding of AP interchain repulsion, likely *via* altering the local dielectric environment or redistributing counterions around the pectin backbone. This shielding effect enables AP chains to approach sufficiently to form extensive hydrogen bonds and hydrophobic associations, while excess NaCl disrupts this electrostatic balance, re-enhances interchain repulsion, and destroys the preformed hydrogel network. Collectively, these findings demonstrate that hydrogen bonding is the dominant driving force for the formation and stabilization of AP–CA composite hydrogels, with hydrophobic interactions providing auxiliary stabilization. The CA-mediated shielding of interchain electrostatic repulsion is a prerequisite for the occurrence of hydrogen bonding and hydrophobic association, and these three effects synergistically drive the spontaneous gelation of the AP–CA system.

### 3.14 Possible mechanism of CA-induced spontaneous gelation of AP

**Fig. 13:**
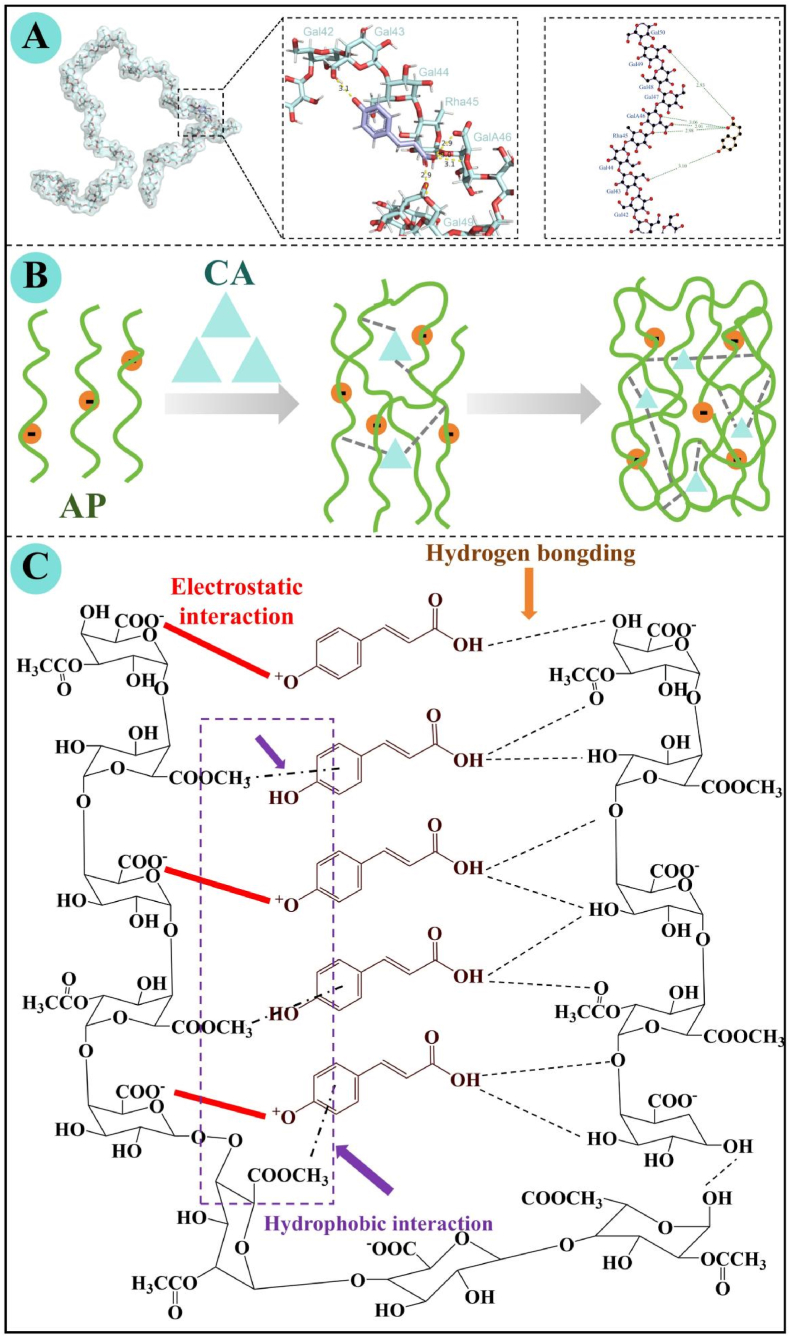
Proposed mechanism of CA-induced spontaneous gelation of AP. (**A**) Molecular docking results of CA with pectin. From left to right: visualization of the CA–pectin docking; three-dimensional interaction; two-dimensional interaction; (**B**) Schematic illustration of the proposed gelation mechanism; (**C**) Proposed molecular binding mode between CA and AP. Note: In panel (A, left), pectin is displayed as a blue stick model with a transparent surface, and CA is shown in purple. Oxygen and hydrogen atoms are colored red and white, respectively. In panel (A, middle), yellow dashed lines indicate hydrogen bonds, with bond lengths shown in Å. In panel (A, right), pectin fragments are colored purple, CA is shown in orange-yellow, and green dashed lines denote hydrogen bonds. Hydrogen bond lengths are presented with two decimal places in the 2D diagram, while the 3D visualization retains only one decimal place, which may result in rounding differences.

Based on the comprehensive results of ζ-potential measurement, FT-IR characterization, rheological analysis, morphological observation and molecular docking, the spontaneous gelation of AP induced by CA is inferred to be a synergistic process driven by electrostatic modulation and multiple non-covalent intermolecular interactions, which promote the association of AP chains and the formation of a continuous three-dimensional network. ζ-potential measurements showed that the absolute ζ-potential of AP–CA mixtures increased initially with rising CA concentration, reflecting the introduction of negatively charged carboxyl and phenolic hydroxyl groups from CA into the system (Fig. S7). After the formation of self-supporting hydrogels, the absolute ζ-potential of the composite hydrogels decreased with increasing CA loading. This trend is consistent with the enhanced association of AP chains, where charged groups are partially embedded within the compact network structure, leading to counterion shielding and reduced surface charge accessibility. These observations indicate that the gelation process is dominated by the intermolecular association of polymer chains, rather than simple electrostatic accumulation. FT-IR analysis revealed a progressive red shift of the O–H stretching peak of AP with increasing CA concentration, which is consistent with the formation of intermolecular hydrogen bonds between the hydroxyl/carboxyl groups of AP and the phenolic hydroxyl groups of CA. Potential hydrophobic interactions between the aromatic rings of CA and the hydrophobic domains of the pectin backbone may also contribute to the intermolecular association of AP chains. SEM imaging and quantitative analysis using AngioToolshowed that the porestructure of the hydrogel network was highly dependent on CA concentration. Moderate CA concentrations resulted in a well-defined honeycomb-like network with high pore connectivity and low lacunarity, while excessive CA (>7 mg/mL) caused local aggregation of AP chains and irregular pore enlargement. These morphological changes are consistent with the trend of enhanced intermolecular association at higher CA concentrations, and align with the rheological properties and mechanical rigidity of the composite hydrogels. Molecular docking studies provide a predictive binding mode between CA and AP chains (Fig. 13A). The calculated binding free energy between CA and the AP fragment was −3.2 kcal/mol. The negative value of binding free energy indicates that the binding process between CA and AP is thermodynamically spontaneous; in general, a lower (more negative) binding free energy corresponds to a more stable intermolecular interaction. The binding free energy obtained here reflects a moderate and stable binding affinity between CA and AP, which is consistent with the moderate non-covalent intermolecular interactions revealed by intermolecular force analysis, and explains why CA can promote the ordered self-assembly of AP chains without causing irreversible over-aggregation. In addition, CA may preferentially bind to the galacturonic acid units (Gal43, Gal44, GalA46, and Gal49) and rhamnose unit (Rha45) of AP fragment. Specifically, CA formed 5 hydrogen bonds with key sugar residues of the AP fragment (including Gal43, GalA46 and Gal49) *via* its carboxyl and phenolic hydroxyl groups, with bond lengths ranging from 2.91 Å to 3.10 Å, forming a stable hydrogen bond network. Meanwhile, extensive hydrophobic interactions were detected between the aromatic ring of CA and hydrophobic sugar residues of AP (including Gal44, Rha45, GalA46 and Gal49), which further stabilized the AP–CA complex. These simulation results are consistent with the enhanced network elasticity, water-holding capacity, and mechanical strength of the composite hydrogels observed in the experiments, providing molecular-level support for the proposed gelation mechanism. Notably, the docking model uses an artificial oligosaccharide fragment, which is a reasonable simplified model of AP but cannot fully replicate the high structural heterogeneity of native AP in real hydrogels. Docking results only predict potential binding between CA and AP's core domain, not all intermolecular interactions in the real system.

Based on the above results, the proposed process of CA-induced AP gelation is illustrated in Fig. 13B and C, and can be described as follows: (i) CA forms non-covalent associations with AP chains *via* hydrogen bonding and potential hydrophobic interactions, forming AP–CA complexes; (ii) progressive intermolecular association of AP–CA complexes drives the formation of a three-dimensional network; and (iii) network consolidation results in the formation of a mechanically robust, elastic hydrogel. Optimal gelation performance is achieved at intermediate CA concentrations, where balanced chain association and network homogeneity are obtained, whereas both insufficient and excessive CA loading impair the structural integrity of the hydrogel. This proposed mechanism integrates electrostatic modulation, non-covalent interactions, and network-level structural reorganization, providing a systematic understanding of CA-induced spontaneous gelation of AP.

Notably, the AP–CA composite hydrogels in this work can be fabricated *via* simple one-step mixing at room temperature, without the need for harsh processing conditions such as thermal polymerization, ultraviolet photo-polymerization, oxidative crosslinking, or freeze-drying, which are widely used in the fabrication of traditional polysaccharide–polyphenol hydrogels. Compared with these conventional approaches, this mild, facile preparation strategy has the advantages of low raw material cost, simplified operation, reduced time and energy consumption, and good scalability for industrial production. Furthermore, the absence of additional chemical crosslinkers or toxic additives preserves the inherent biocompatibility of natural AP and CA. A similar mild gelation strategy *via* simple mixing at ambient conditions has also been reported in sodium alginate–gallic acid hydrogel systems (Chen et al., 2024).

## 4 Conclusion

This study establishes a novel, mild, and clean-label strategy to achieve spontaneous gelation of native low-methoxyl apple pectin (AP, DM 30%–40%) solely mediated by p-coumaric acid (CA), eliminating the dependence on sucrose, calcium ions, or thermal treatment that are indispensable for conventional pectin gelation systems. The three preset core research objectives have been fully accomplished, with key findings summarized as follows: First, the gelation behavior of the AP–CA system was systematically characterized, with the critical gelation concentration of CA precisely defined as 4.0 mg/mL under the experimental acidic condition (pH 3.0). Stable self-supporting hydrogels were formed at CA concentrations of 4–9 mg/mL, and the microstructure, viscoelasticity, and water retention properties of the hydrogels can be flexibly tuned *via* adjustment of CA concentration. The composite hydrogels exhibit pronounced shear-thinning behavior, excellent syringeability, and long-term shape retention after extrusion, which meets the processing requirements of injectable food systems. Second, the functional properties of the AP–CA composite hydrogels were evaluated. Incorporation of CA endows the hydrogels with robust antioxidant activity (>95% ABTS radical scavenging) and broad-spectrum antibacterial effects against common foodborne pathogens, while maintaining favorable compatibility with *L. plantarum* across the tested CA concentration range. These functional properties confer significant application potential of the hydrogel system in the development of low-sugar functional foods and probiotic delivery carriers. Third, the molecular mechanism underlying CA-induced spontaneous gelation of AP was elucidated through multi-scale structural characterization, intermolecular force analysis, and molecular docking simulation. Hydrogen bonding between CA and AP chains is identified as the dominant driving force for the formation and stabilization of the three-dimensional gel network, with hydrophobic interactions providing auxiliary stabilization. CA-mediated shielding of interchain electrostatic repulsion serves as a prerequisite for the occurrence of ordered intermolecular assembly. The moderate non-covalent interactions derived from the unique structural features of CA are the core factor enabling its AP gelation-inducing capacity, which is not achievable with other common phenolic acids. The core innovation of this work lies in the first realization of spontaneous room-temperature gelation of native pectin solely *via* non-covalent interactions with CA, completely free of exogenous additives or harsh processing conditions. This strategy breaks the inherent limitations of conventional pectin gelation systems, and provides a new theoretical and technical basis for the development of clean-label, low-sugar pectin-based functional materials. It should be noted that this study is limited to laboratory-scale physicochemical and functional characterization of the hydrogel system under acidic conditions (pH 3.0). Future research will focus on verifying the universality of this gelation mechanism in pectin from different sources and structural features, evaluating the performance of the hydrogels in real food matrices such as thermal processing (Fig. S8), and investigating the probiotic encapsulation and delivery behavior of the system in simulated gastrointestinal digestion.

## CRediT authorship contribution statement

Lanlan Hu: Conceptualization, Data curation, Formal analysis, Resources, Writing – original draft. Yifei Bai: Investigation, Methodology, Validation. Guanglei Li: Project administration, Resources, Software. Ibrahim Khalifa: Software, Supervision, Validation. Benguo Liu: Resources, Validation, Writing – review & editing. Hao Zhang: Formal analysis, Project administration, Writing – review & editing. Yangyang Jia: Data curation, Funding acquisition, Project administration, Supervision, Writing – review & editing.

## Declaration of competing interest

The authors declare that they have no known competing financial interests or personal relationships that could have appeared to influence the work reported in this paper.

## Data Availability

Data will be made available on request.

## Abbreviation list

ABTS: 2,2′-azino-bis(3-ethylbenzothiazoline-6-sulfonic acid); AP: apple pectin; CA: p-coumaric acid; CPMG: Carr-Purcell-Meiboom-Gill pulse sequence; DPPH: 2,2-diphenyl-1-picrylhydrazyl; FTIR: Fourier transform infrared spectroscopy; *G′*: storage modulus; *G″*: loss modulus; *G∗*: complex modulus; HG: homogalacturonan; HMP: high methoxyl pectin; KBr: potassium bromide; LF-NMR: low-field nuclear magnetic resonance; LMP: low methoxyl pectin; LVR: linear viscoelastic region; MRS: de Man Rogosa Sharp; NaCl: sodium chloride; NLVR: non-linear viscoelastic region; R^2^: correlation coefficients; RG-I: rhamnogalacturonan-I; RG-II: rhamnogalacturonan-II; SDS: sodium dodecyl sulfate; SR: syneresis rate; TPA: Texture Profile Analysis; WHC: water holding capacity; XRD: X-ray diffraction; Δ*E*∗: chromaticity difference.

## References

[bib1] Ahn S., Halake K., Lee J. (2017). Antioxidant and ion-induced gelation functions of pectins enabled by polyphenol conjugation. Int. J. Biol. Macromol..

[bib2] Chen Q., Yang Z.-R., Du S., Chen S., Zhang L., Zhu J. (2024). Polyphenol-sodium alginate supramolecular injectable hydrogel with antibacterial and anti-inflammatory capabilities for infected wound healing. Int. J. Biol. Macromol..

[bib3] Derebasi B.N., Davran Bulut S., Aksoy Erden B., Sadeghian N., Taslimi P., Celebioglu H.U. (2024). Effects of p-coumaric acid on probiotic properties of *Lactobacillus acidophilus* LA-5 and Lacticaseibacillus rhamnosus GG. Arch. Microbiol..

[bib4] Eberhardt J., Santos-Martins D., Tillack A.F., Forli S. (2021). AutoDock vina 1.2. 0: new docking methods, expanded force field, and python bindings. J. Chem. Inf. Model..

[bib5] Ettoumi F.-e., Huang H., Xu Y., Wang L., Ru Q., Hu Y., Zou L., Luo Z. (2024). Supramolecular assembly of dual crosslinked nanocomposite polysaccharides hydrogel: integration of injectable, self-healing, and pH-responsive platform for sustained delivery of polyphenols. Food Hydrocoll..

[bib6] Fang Q., Xu T., Su R., Dai S., Wang J., Zhu W., Yang B., Tong X., Wang H., Jiang L. (2025). Composite gel based on κ-carrageenan-soybean isolate protein/soy protein fibrils: focus on structural differences and gel properties. Int. J. Biol. Macromol..

[bib7] Feng S., Yi J., Ma Y., Bi J. (2023). The role of amide groups in the mechanism of acid-induced pectin gelation: a potential pH-sensitive hydrogel based on hydrogen bond interactions. Food Hydrocoll..

[bib8] Gong W., Huang H.-b., Wang X.-c., He W.-y., Hu J.-n. (2022). Coassembly of fiber hydrogel with antibacterial activity for wound healing. ACS Biomater. Sci. Eng..

[bib9] Grant O.C., Wentworth D., Holmes S.G., Kandel R., Sehnal D., Wang X., Xiao Y., Sheppard P., Grelsson T., Coulter A. (2025). Generating 3D models of carbohydrates with GLYCAM-web. bioRxiv.

[bib10] Hazrati Z., Madadlou A. (2021). Gelation by bioactives: characteristics of the cold-set whey protein gels made using gallic acid. Int. Dairy J..

[bib11] Hu X., Meng Z. (2025). Plant-based yolk alternatives based on alginate-chitosan and gellan gum-chitosan double hydrogel network using reverse spherification technology. Food Chem..

[bib12] Huang Y., Condict L., Richardson S.J., Brennan C.S., Kasapis S. (2023). Exploring the inhibitory mechanism of p-coumaric acid on α-amylase *via* multi-spectroscopic analysis, enzymatic inhibition assay and molecular docking. Food Hydrocoll..

[bib13] Karppanen H., Halahlah A., Kilpeläinen P.O., Mikkonen K.S., Ho T.M. (2023). Gel characteristics of low-acetyl spruce galactoglucomannans. Carbohydr. Polym..

[bib14] Kazemi-Taskooh Z., Varidi M. (2021). Designation and characterization of cold-set whey protein-gellan gum hydrogel for iron entrapment. Food Hydrocoll..

[bib15] Kong S., Ma X., Zhen S., Liu Y., Sun F., Yang N. (2025). Elucidating the effect of chitosan microgel characteristics on the large amplitude oscillatory shear (LAOS) behavior of their stabilized high internal phase emulsions using the sequence of physical processes (SPP) approach and comparison with mayonnaise. Int. J. Biol. Macromol..

[bib16] Kong Y., Lin S., Chen S., Jing L., Liu Z., Wu M., Yu X., Fu C., Wang J., Huang D. (2025). Pumpkin seed protein-based hydrogel as gelatin mimics and edible inks in 3D-Printed food. Food Hydrocoll..

[bib17] Kopač T., Abrami M., Grassi M., Ručigaj A., Krajnc M. (2022). Polysaccharide-based hydrogels crosslink density equation: a rheological and LF-NMR study of polymer-polymer interactions. Carbohydr. Polym..

[bib18] Li H., Rao J., Chen B. (2023). Tyramine modification of high and low methoxyl pectin: physicochemical properties, antioxidant activity, and gelation behavior. Food Hydrocoll..

[bib19] Li Q., Xu M., Xie J., Su E., Wan Z., Sagis L.M., Yang X. (2021). Large amplitude oscillatory shear (LAOS) for nonlinear rheological behavior of heterogeneous emulsion gels made from natural supramolecular gelators. Food Res. Int..

[bib20] Liu Y., Dong L., Li Y., Chen Q., Wang L., Farag M.A., Liu L., Zhan S., Wu Z., Liu L. (2023). Soy protein isolate-citrus pectin composite hydrogels induced by TGase and ultrasonic treatment: potential targeted delivery system for probiotics. Food Hydrocoll..

[bib21] Liu Y., Teng J., Huang R., Zhao W., Yang D., Ma Y., Wei H., Chen H., Zhang J., Chen J. (2024). Injectable plant-derived polysaccharide hydrogels with intrinsic antioxidant bioactivity accelerate wound healing by promoting epithelialization and angiogenesis. Int. J. Biol. Macromol..

[bib22] Ma H., Axi Y., Lu Y., Dai C., Huang S., Kong Z., Jimo R., Li H., Chen G., Li P. (2024). A dual network cross-linked hydrogel with multifunctional *Bletilla striata* polysaccharide/gelatin/tea polyphenol for wound healing promotion. Int. J. Biol. Macromol..

[bib23] Ma Q., Xu Q., Chen N., Zeng W. (2024). Gel properties of *Nicandra physalodes* (Linn.) gaertn. seeds polysaccharides with tea polyphenols and its application. Food Chem. X.

[bib24] Ma W., Yuan C., Cui B., Gao T., Guo L., Yu B., Zhao M., Zou F. (2024). Highly-branched cyclic dextrin for improvement in mechanical properties and freeze-thaw stability of κ-carrageenan gels. Food Hydrocoll..

[bib25] Maiz-Fernández S., Barroso N., Pérez-Álvarez L., Silván U., Vilas-Vilela J.L., Lanceros-Mendez S. (2021). 3D printable self-healing hyaluronic acid/chitosan polycomplex hydrogels with drug release capability. Int. J. Biol. Macromol..

[bib26] Mamet T., Yao F., Li K., Li C. (2017). Persimmon tannins enhance the gel properties of high and low methoxyl pectin. LWT.

[bib27] Matheus J., Alegria M.J., Nunes M.C., Raymundo A. (2024). Algae-boosted chickpea hummus: improving nutrition and texture with seaweeds and microalgae. Foods.

[bib28] Petkowicz C.L., Williams P.-A. (2020). Pectins from food waste: characterization and functional properties of a pectin extracted from broccoli stalk. Food Hydrocoll..

[bib29] Qiu G., Xu Z., Wu J.-Y., Li C., Hu Z., Huang R., Zhong Y., Liu X. (2025). Litchi polyphenols and carboxylated cellulose nanofiber synergistically improve the gel properties of κ-carrageenan gels: insight from rheology, morphology and interaction computational simulation. Food Hydrocoll..

[bib30] Senan A.M., Muhammed M.T., Akkoc S., Alhag S.K., Al-Shahari E.A., Al-Shuraym L.A. (2025). Modification of ionic liquid and lactoferrin-based small molecules as potential therapeutics against SARS-CoV-2: molecular docking disclosed the predictable results. J. Mol. Struct..

[bib31] Shang L., Wu C., Wang S., Wei X., Li B., Li J. (2021). The influence of amylose and amylopectin on water retention capacity and texture properties of frozen-thawed konjac glucomannan gel. Food Hydrocoll..

[bib32] Srichuwong S., Isono N., Jiang H., Mishima T., Hisamatsu M. (2012). Freeze–thaw stability of starches from different botanical sources: correlation with structural features. Carbohydr. Polym..

[bib33] Tang S., Chi K., Xu H., Yong Q., Yang J., Catchmark J.M. (2021). A covalently cross-linked hyaluronic acid/bacterial cellulose composite hydrogel for potential biological applications. Carbohydr. Polym..

[bib34] Tong C., Huang C., Lai H., Li K., Zeng X., Wen C., Wu C., Pang J. (2024). Effect of carboxylated cellulose nanocrystals on konjac glucomannan/κ-carrageenan composite hydrogels. Food Hydrocoll..

[bib35] Trott O., Olson A.J. (2010). AutoDock vina: improving the speed and accuracy of docking with a new scoring function, efficient optimization, and multithreading. J. Comput. Chem..

[bib36] Uslu H., Das B., Dagdogen H.A., Santur Y., Yılmaz S., Turkoglu I., Das R. (2025). Discovery of new anti-HIV candidate molecules with an AI-based multi-stage system approach using molecular docking and ADME predictions. Chemometr. Intell. Lab. Syst..

[bib37] Vityazev F.V., Khramova D.S., Saveliev N.Y., Ipatova E.A., Burkov A.A., Beloserov V.S., Belyi V.A., Kononov L.O., Martinson E.A., Litvinets S.G. (2020). Pectin–glycerol gel beads: preparation, characterization and swelling behaviour. Carbohydr. Polym..

[bib38] Wang H., Ke L., Ding Y., Rao P., Xu T., Han H., Zhou J., Ding W., Shang X. (2022). Effect of calcium ions on rheological properties and structure of *Lycium barbarum* L. polysaccharide and its gelation mechanism. Food Hydrocoll..

[bib39] Wang Z., Xiao N., Guo S., Tian X., Ai M. (2024). Tea polyphenol-mediated network proteins modulate the NaOH-heat induced egg white protein gelling properties. Food Hydrocoll..

[bib40] Xie F., Liu M., Feng X., He Z., Chen Q., Zhou J., Cai J. (2025). Tannic acid one-step induced quaternized chitin-based edible and easy-cleaning coatings with multifunctional preservation for perishable products. Food Hydrocoll..

[bib41] Xu S., Liu H., Yan J., Wang C., Lai B., Wu H. (2024). Interaction mechanism and binding mode between different polyphenols and gellan gum. Food Hydrocoll..

[bib42] Xu Y., Pan X., Zhao W., Luo Q., Lao F., Guo X., Pang X., Xiao Z., Wu J. (2025). Insights into the non-covalent interaction between muskmelon peel pectin and selected C9 aldehydes by the application of multiple spectroscopy and molecular docking. Food Hydrocoll..

[bib43] Yan W., Jia X., Zhang Q., Chen H., Zhu Q., Yin L. (2021). Interpenetrating polymer network hydrogels of soy protein isolate and sugar beet pectin as a potential carrier for probiotics. Food Hydrocoll..

[bib44] Yang C., Guo C., Gao B., Kurek M.A., Niu Y., Yu L. (2025). Physical interaction between gallic acid and *monascus* pigments enhances mechanical strength and pigment protection of pectin-gelatin hydrogel carriers. Food Hydrocoll..

[bib45] Yang X., Nisar T., Liang D., Hou Y., Sun L., Guo Y. (2018). Low methoxyl pectin gelation under alkaline conditions and its rheological properties: using NaOH as a pH regulator. Food Hydrocoll..

[bib46] You S., Huang Y., Mao R., Xiang Y., Cai E., Chen Y., Shen J., Dong W., Qi X. (2022). Together is better: poly (tannic acid) nanorods functionalized polysaccharide hydrogels for diabetic wound healing. Ind. Crop. Prod..

[bib47] Zhang X., Mu Y., Zhao L., Hong Y., Shen L. (2024). Self-healing, antioxidant, and antibacterial *Bletilla striata* polysaccharide-tannic acid dual dynamic crosslinked hydrogels for tissue adhesion and rapid hemostasis. Int. J. Biol. Macromol..

[bib48] Zhong Y., Zeng S., Lv Y., Lv W., Xiao H., Sheng S. (2024). Effect of guar gum on the rheological properties, microstructure and 3D printing performance of egg yolk powder-potato starch composite gel. Food Hydrocoll..

[bib49] Zhou Z., Xiao J., Guan S., Geng Z., Zhao R., Gao B. (2022). A hydrogen-bonded antibacterial curdlan-tannic acid hydrogel with an antioxidant and hemostatic function for wound healing. Carbohydr. Polym..

[bib50] Zhu Z., Wu Y., Zhong Y., Zhang H., Zhong J. (2024). Development, characterization and *Lactobacillus plantarum* encapsulating ability of novel C-phycocyanin-pectin-polyphenol based hydrogels. Food Chem..

