# Supplementary material for: Coumaric acid-induced spontaneous gelation of apple pectin with controllable structure and multifunctional properties
Source: Curr Res Food Sci. 2026 May 18;12:101443. doi: 10.1016/j.crfs.2026.101443 (PMC13217479; doi:10.1016/j.crfs.2026.101443)
Supplement: Multimedia component 1 [file mmc1.docx]

**Coumaric acid-induced spontaneous gelation of apple pectin with controllable structure and multifunctional properties**

Lanlan Hu ^a,b^, Yifei Bai ^a^, Guanglei Li ^a^, Ibrahim Khalifa ^c,d^, Benguo Liu ^a^,

Hao Zhang ^a,b,*^, Yangyang Jia ^a,*^

a School of Food Science, Henan Institute of Science and Technology, Xinxiang, 453003, China.

b Shaanxi Research Institute of Agricultural Products Processing Technology, Xi’an, 710021, China.

c Food Technology Department, Faculty of Agriculture, Benha University, Moshtohor, 13736, Egypt.

d Department of Food Science, College of Agriculture and Veterinary Medicine, United Arab Emirates University, Al-Ain 15551, United Arab Emirates.

* Corresponding author.

1. mail address: [Jiayangyang18@163.com;](mailto:Jiayangyang18@163.com;) [Zhanghao@hist.edu.cn](mailto:Zhanghao@hist.edu.cn)

**Supplementary materials**


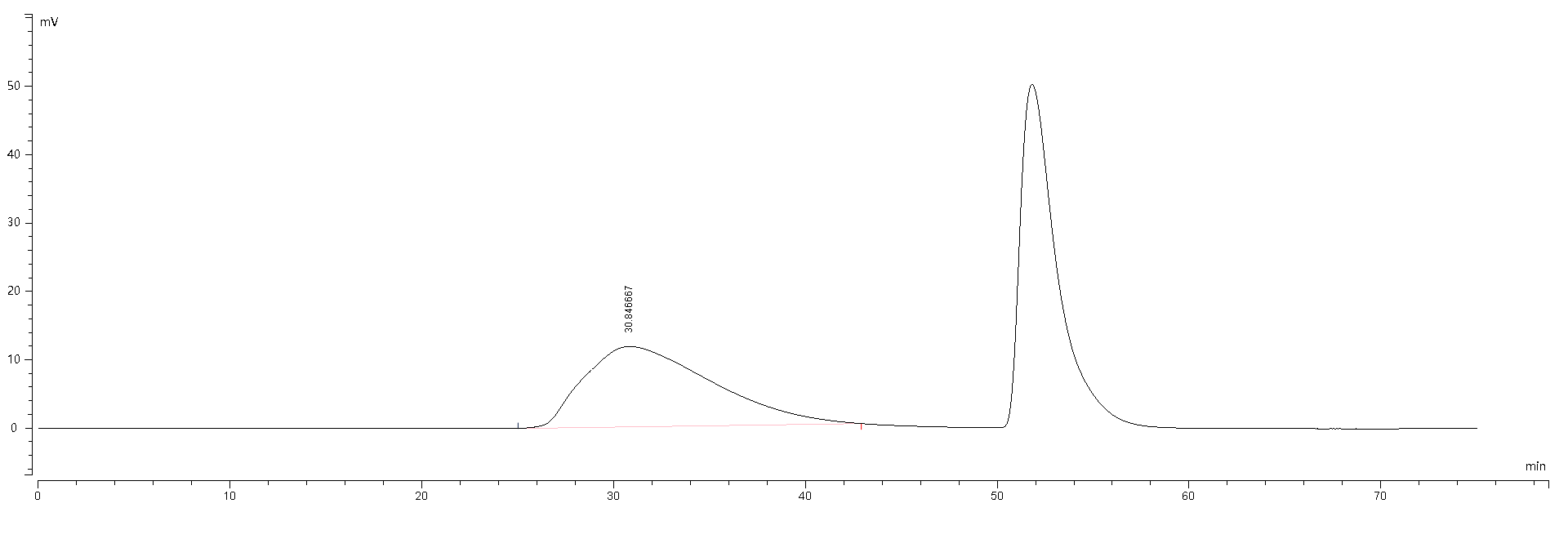


**Fig. S1** HPGPC chromatogram of AP.


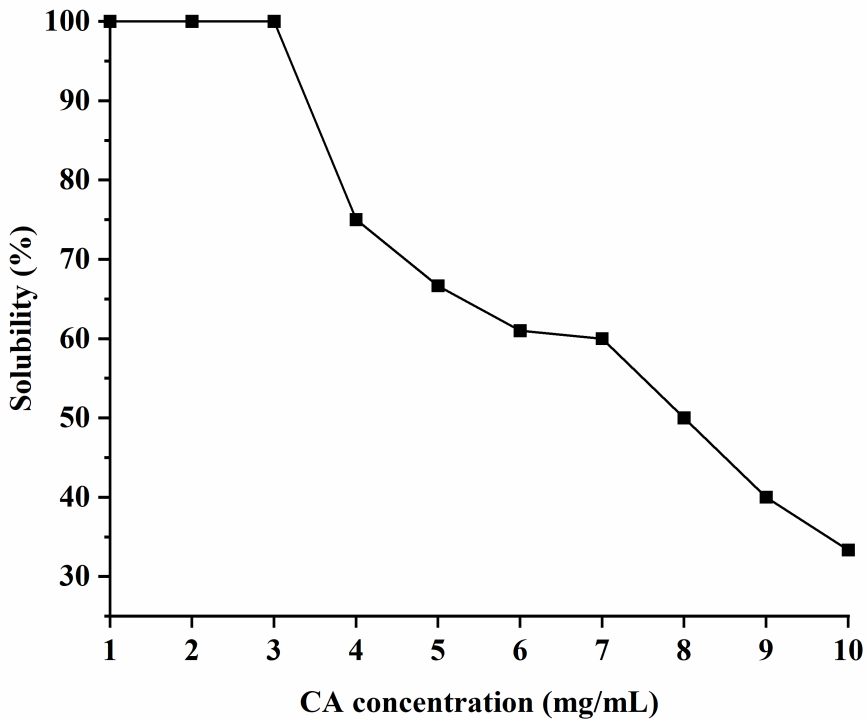


**Fig. S2** Solubility curve of CA in aqueous system.


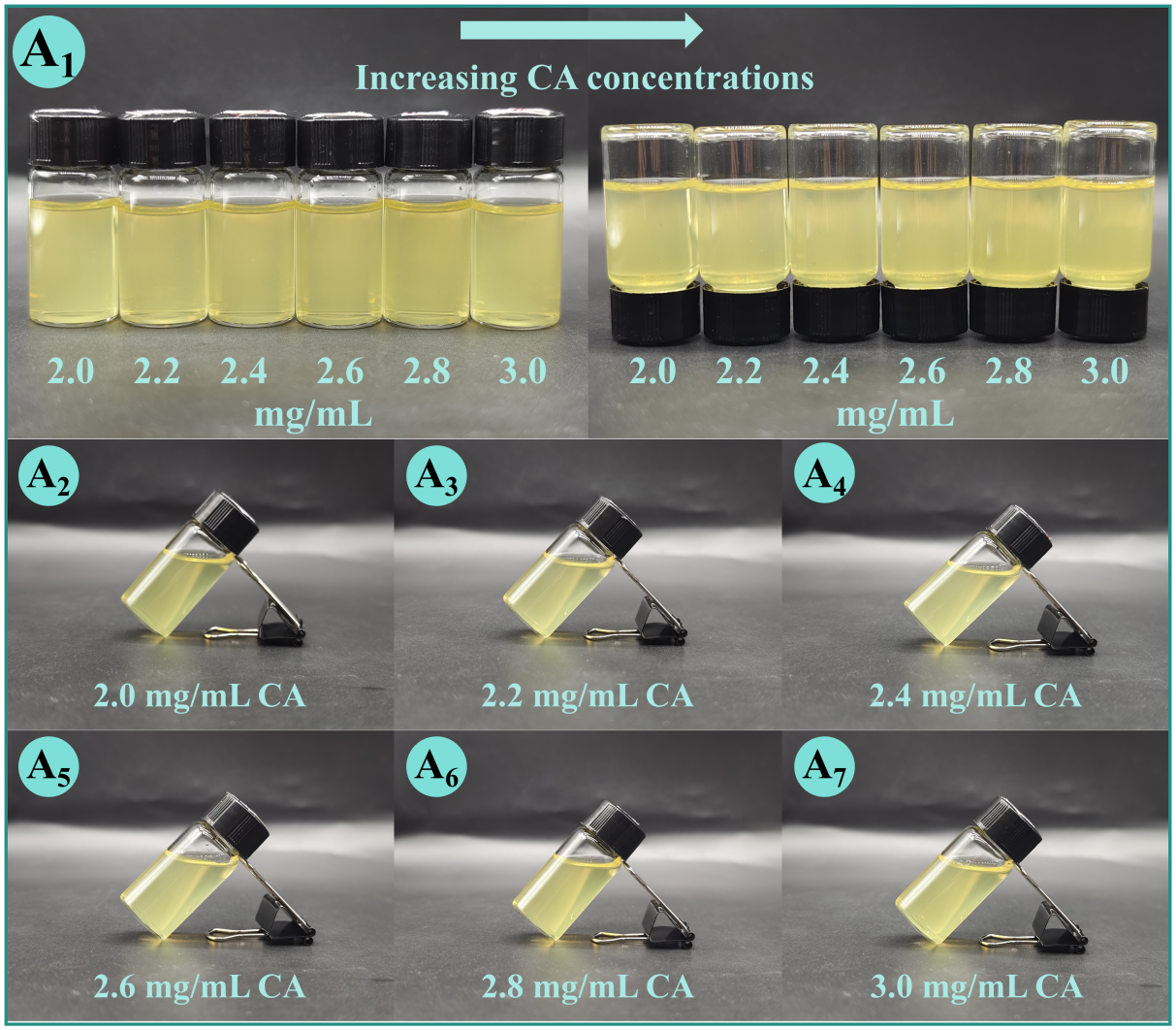


**Fig. S3** Visual appearance of AP–CA composite hydrogels prepared with CA concentrations ranging from 2.0 to 3.0 mg/mL at 0.2 mg/mL intervals: (**A_1_**) samples under normal and inverted placement; (**A_2_–A_7_**) samples under 45° tilted view


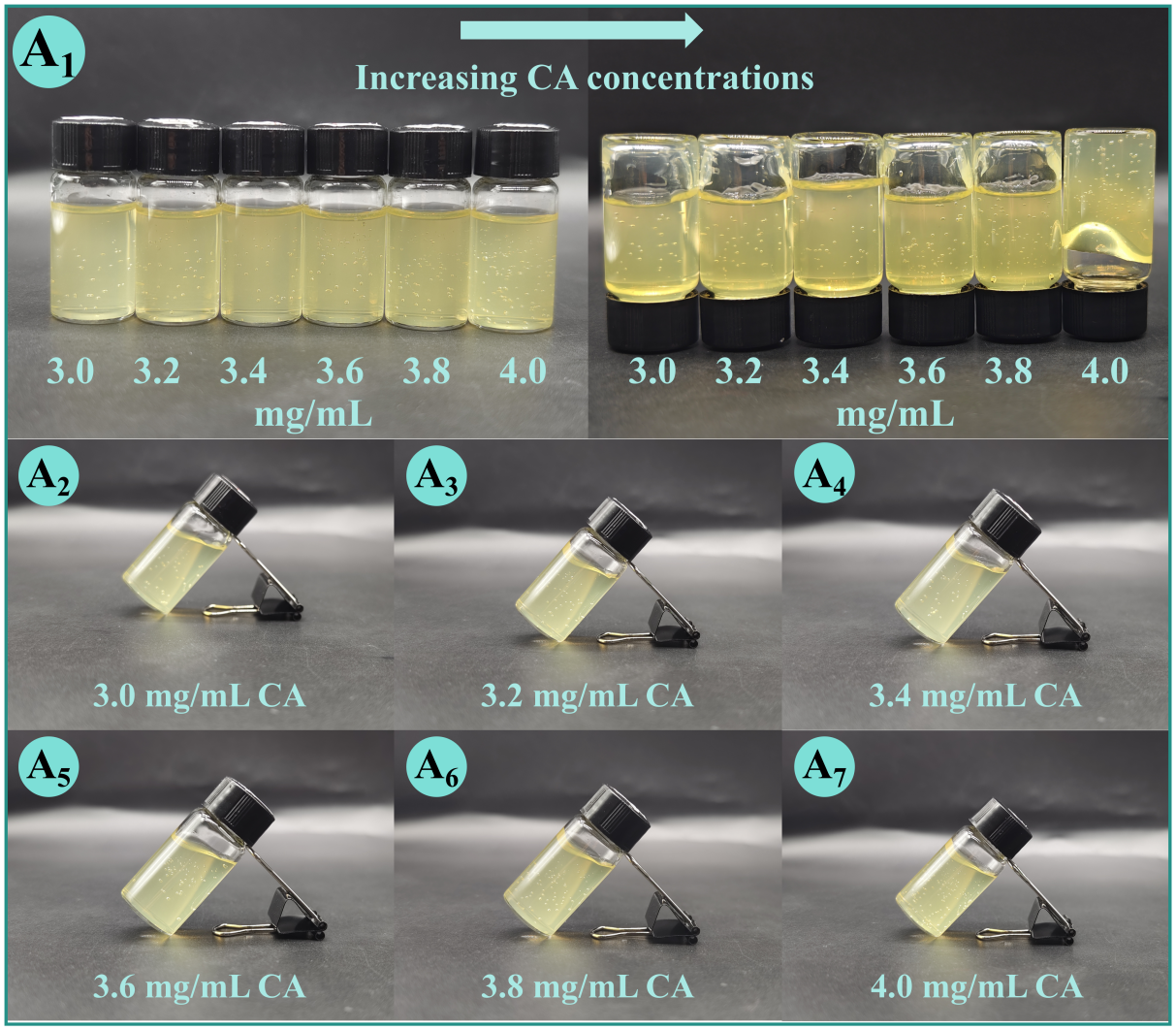


**Fig. S4** Visual appearance of AP–CA composite hydrogels prepared with CA concentrations ranging from 3.0 to 4.0 mg/mL at 0.2 mg/mL intervals: (**A_1_**) samples under normal and inverted placement; (**A_2_–A_7_**) samples under 45° tilted view


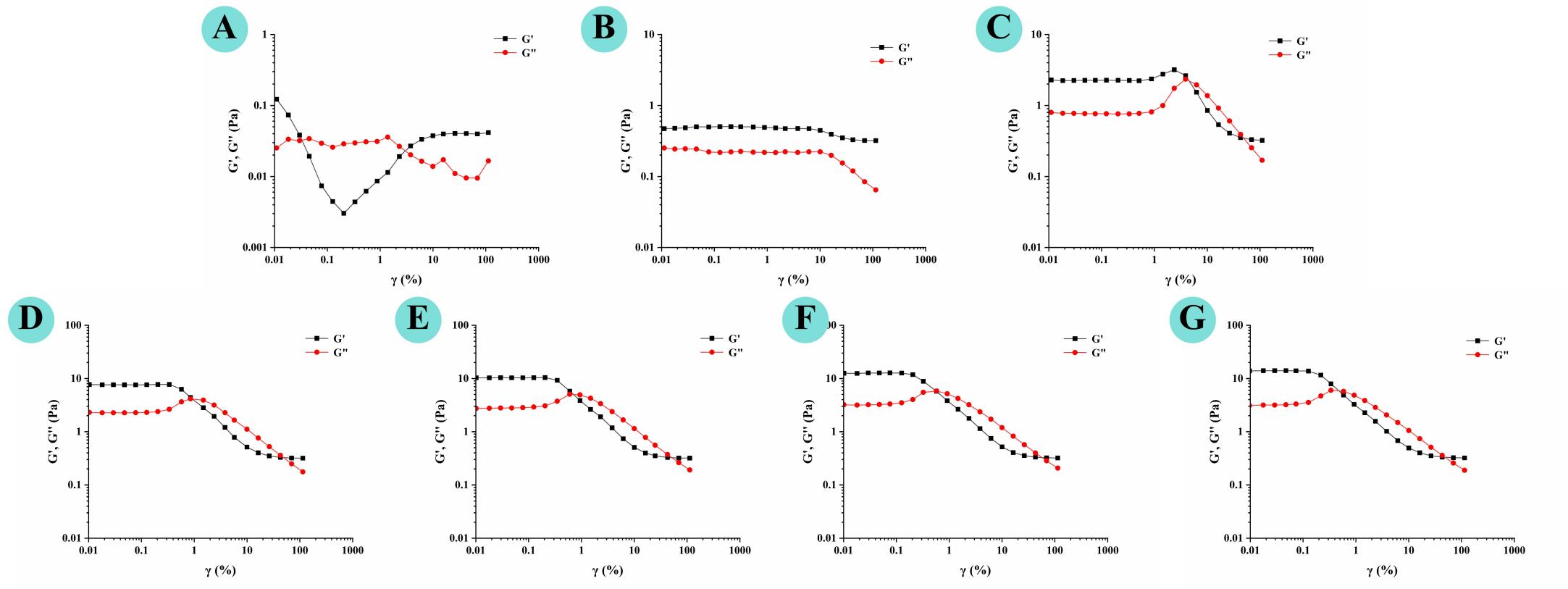


**Fig. S5 (A–G**) *G′* and *G″* values under strain sweep at CA concentrations of 0, 3, 4, 6, 7, 8, and 9 mg/mL, respectively.

**
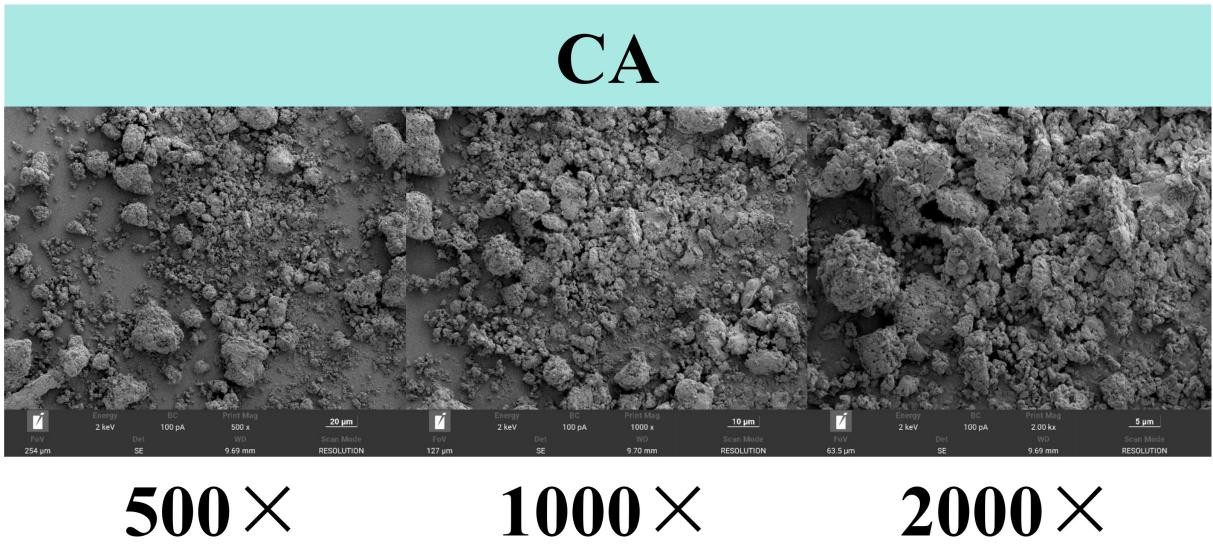
**

**Fig. S6** SEM micrographs of dried CA.


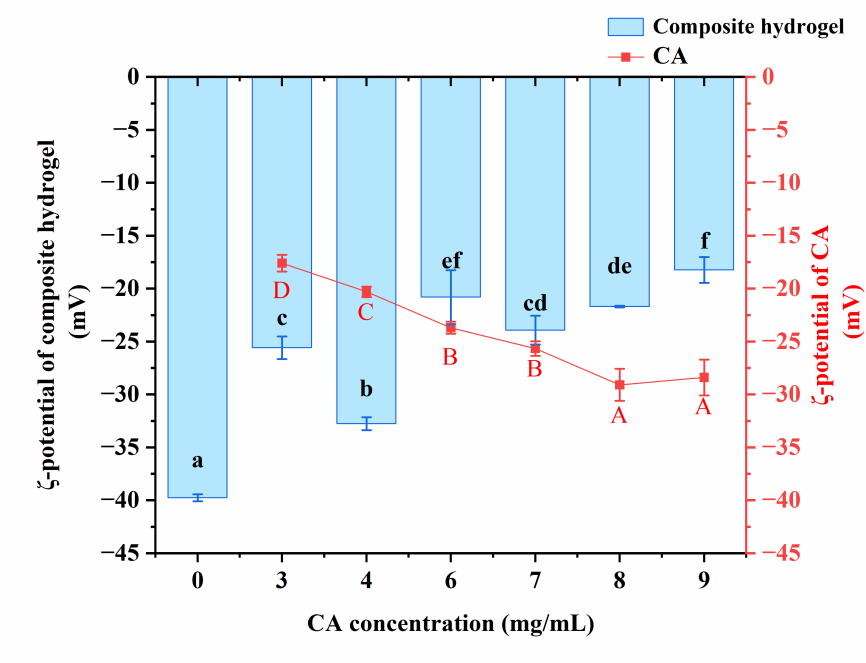


**Fig. S7** ζ-potential of CA and AP–CA composite hydrogels.


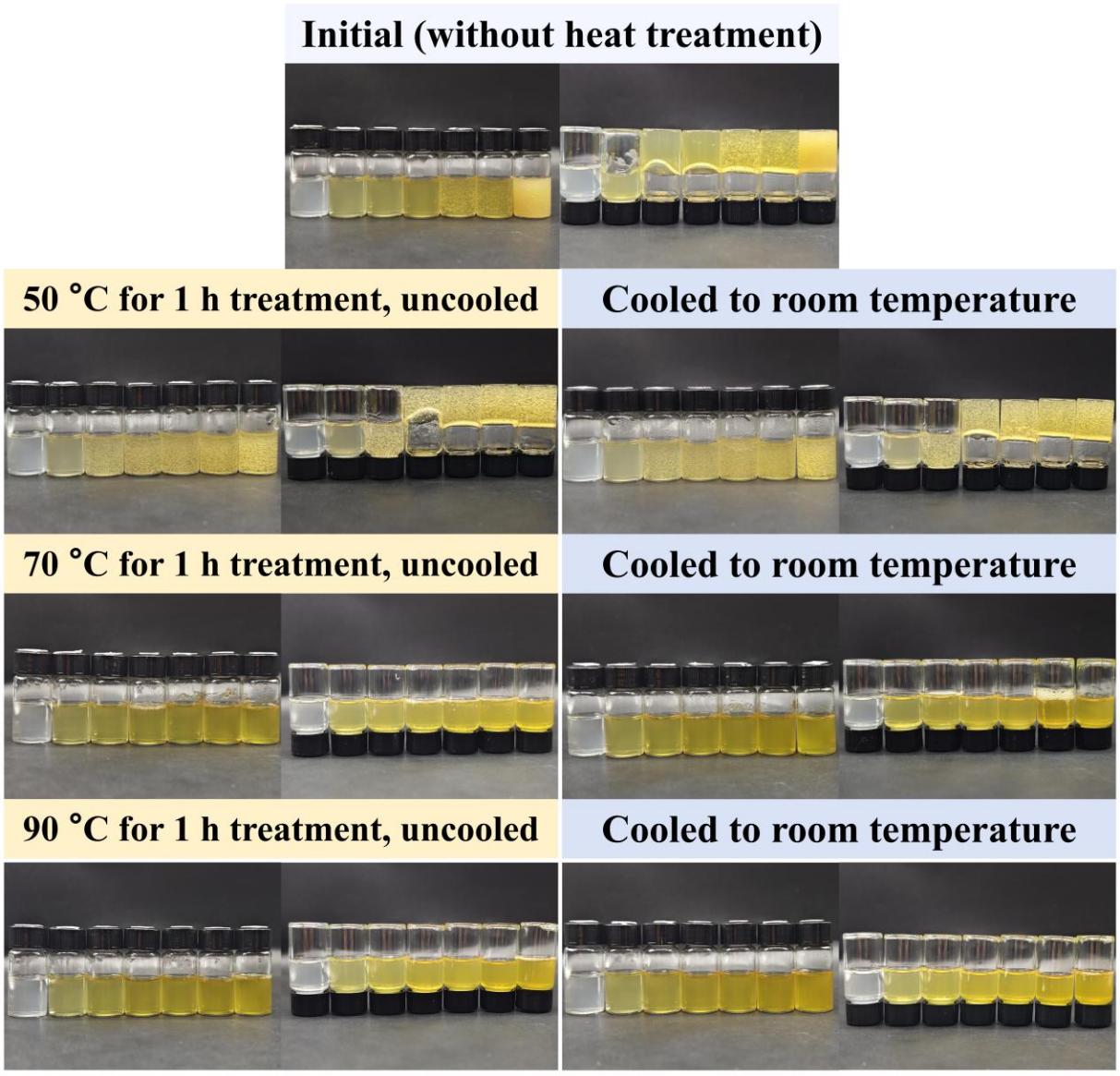


**Fig. S8** State change of AP–CA composite hydrogels before and after heat treatment

**Table S1** Molecular weight information of AP

|  | **RT (min)** | **lgMp** | **lgMw** | **lgMn** | **Mp** | **Mw** | **Mn** |
| --- | --- | --- | --- | --- | --- | --- | --- |
| Peak Start | 25.4 | 6.2 | 6.2 | 6.2 | 1584163 | 1565237 | 1509593 |
| Peak Maximum | 30.847 | 5.5 | 5.5 | 5.5 | 326196 | 323108 | 314369 |
| Peak End | 43 | 4.0 | 4.0 | 4.0 | 9598 | 9561 | 9486 |

**Table S2** Quantitative parameters extracted from SEM images of AP–CA composite hydrogels using AngioTool

|  | **Vessel percentage area (%)** | **Total Number of Junctions** | **Mean E Lacunarity** |
| --- | --- | --- | --- |
| Control (AP dispersion) | 24.66 ± 2.57a | 289.50 ± 82.63a | 0.27±0.06a |
| 3 mg/mL CA | 35.13 ± 2.46bc | 527.67 ±82.04bc | 0.15±0.02bcd |
| 4 mg/mL CA | 42.27 ± 1.99d | 704.33 ±66.01d | 0.11±0.01b |
| 6 mg/mL CA | 36.72 ± 1.22c | 586.33 ± 40.56cd | 0.14±0.01bd |
| 7mg/mL CA | 34.28 ± 3.78bc | 498.33 ±99.23bc | 0.16±0.03bc |
| 8 mg/mL CA | 30.08 ± 3.21b | 422.33 ± 48.56b | 0.19±0.03c |
| 9 mg/mL CA | 32.03 ± 4.16bc | 511.00 ±55.87bc | 0.17±0.03bc |

Note: Different letters in the same column indicate significant differences (*p* < 0.05).
